# Dynamic Processes of Rhenium Polyhydride Complexes

**DOI:** 10.3390/molecules27155017

**Published:** 2022-08-07

**Authors:** Datta V. Naik, Gregory A. Moehring

**Affiliations:** Department of Chemistry and Physics, Monmouth University, West Long Branch, NJ 07764, USA

**Keywords:** rhenium, polyhydride, dynamic processes, line shape fitting, proton exchange, pseudorotation, steric inversion, turnstile exchange

## Abstract

Studies have demonstrated that high-coordination-number rhenium polyhydride complexes are precursors to catalysts that transform a variety of organic molecules. While rhenium polyhydride complexes lead to active catalysts, little has been reported on the mechanisms for the transformations. High-coordination-number rhenium polyhydride complexes exhibit several dynamic processes that make characterizations of the chemical properties for individual atoms difficult, at best, for room-temperature solutions. This review describes what is known of the dynamic processes that occur at high-coordination-number rhenium polyhydride complexes and how that knowledge may lead to the design of catalytic precursors in which the chemical properties of individual atoms can be more readily identified in room-temperature solutions.

## 1. Introduction

Rhenium polyhydride complexes (rhenium complexes supported by four or more hydride ligands) have been the focus of numerous studies. The large number of reports on rhenium polyhydride complexes reflect several factors: (1) convenient synthetic routes into such complexes [1,2,3,4]; (2) the relative stability of such complexes, which makes for convenient materials to work with; (3) intrinsically interesting aspects of rhenium polyhydride complexes such as the homoleptic dianion, (ReH_9_)^2−^ or the presence of dihydrogen as a ligand on a metal center with multiple hydride ligands [5,6,7,8,9,10]; (4) the technical challenge of characterizing a dihydrogen ligand in the presence of multiple hydride ligands bound to a metal center [11,12,13,14,15,16,17]; (5) the convenient ability to prepare mixed-metal polyhydride complexes and examine the properties of such complexes [18,19,20,21]; (6) an interest in understanding the dynamic properties and solution behavior of high coordination metal centers stabilized by several hydride ligands [22,23,24,25,26,27,28,29,30,31,32,33,34,35,36,37,38]; and (7) the demonstrated ability for such complexes to serve as precatalysts for the transformation of organic molecules [39,40,41,42,43,44,45,46,47,48]. The latter property, the ability of rhenium polyhydride complexes to form catalysts for organic transformations, is the most enduring interest in rhenium polyhydride complexes. The transformations of rhenium polyhydride complexes from precatalyst to catalyst and the catalytic cycles for organic transformations, however, are generally not well described in the rhenium polyhydride literature. The dynamic nature of the ligands on these high-coordination-number metal centers stabilized by multiple hydride ligands (often described as “fluxionality”) contributes significantly to the inability to describe, in detail, the catalytic cycles associated with most rhenium polyhydride precatalysts.

**Figure 1:**
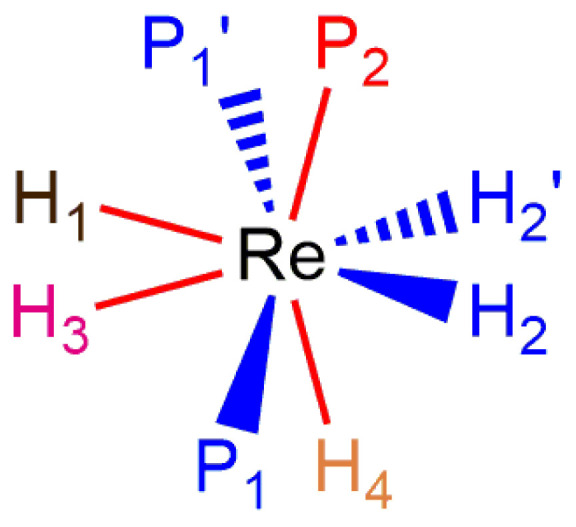
A representation of the arrangement of atoms bound to rhenium in the complex ReH_5_(PPh_3_)_3_. Blue bonds are used to indicate atoms that lie on a horizontal trapezoid. Red bonds are used to depict atoms that lie on a vertical trapezoid. The colors of the atom symbols are used to indicate chemical equivalence (P1 and P1′ or H2 and H2′) or chemically unique atoms. The representation is based on the results of an X-ray diffraction analysis of the complex [14].

**Figure 2:**
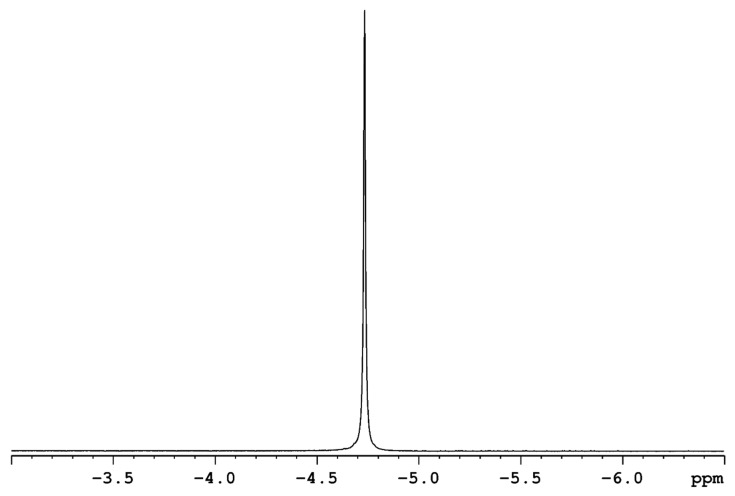
The room-temperature ^1^H-{^31^P} NMR hydride resonance for ReH_5_(PPh_3_)_3_ measured in a solution of d_8_-toluene.

**Figure 3:**
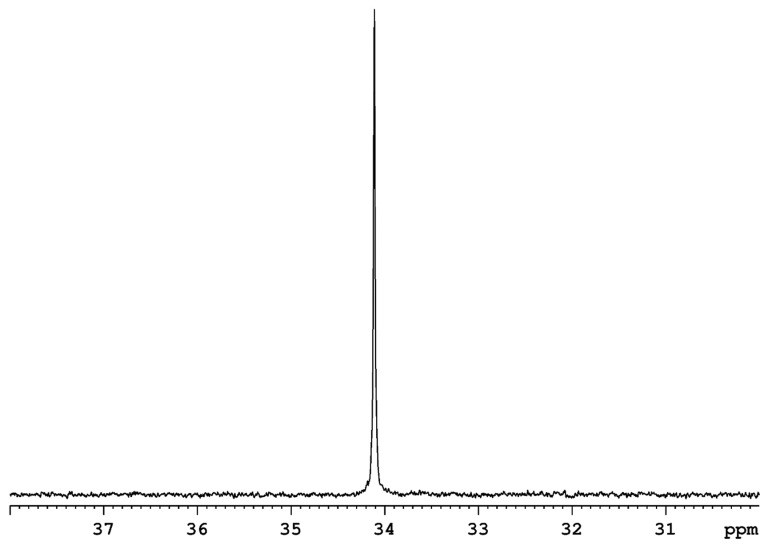
The room-temperature ^31^P-{^1^H} NMR spectrum of ReH_5_(PPh_3_)_3_ measured in a d_8_-toluene solution.

Rhenium polyhydride complexes have long been described as fluxional [49]. The fluxional description of rhenium polyhydride complexes is due to the deceptively simple room-temperature NMR characteristics of such complexes. A rhenium polyhydride complex that was first reported in 1969, ReH_5_(PPh_3_)_3_ [1], provides an excellent example of the deceptively simple room-temperature NMR characteristics of such complexes. In the solid state, ReH_5_(PPh_3_)_3_ has three unique hydride ligands and one pair of chemically equivalent hydride ligands (Figure 1) [14]. The solid-state structure of ReH_5_(PPh_3_)_3_ also has a pair of chemically equivalent phosphorus atoms and one unique phosphorus atom. In room-temperature solutions, only a single ^1^H-{^31^P} NMR resonance is observed (Figure 2) and a single ^31^P-{^1^H} NMR resonance (Figure 3). The simple room-temperature NMR spectra of ReH_5_(PPh_3_)_3_ and similar complexes are due to low-activation-energy dynamic processes involving the hydride ligands and, in nearly all cases, heavier ligands, which allow for fast exchange of atoms between different environments on the NMR time scale in room-temperature solutions. This review aims to summarize what is known about the dynamic processes of rhenium polyhydride complexes. This review will begin with a brief introduction of the catalytic transformations that have been reported to occur at rhenium polyhydride complexes (Section 2), a general description of the structures most common to rhenium polyhydride complexes (Section 3), and a description of the techniques used to characterize the dynamic processes of rhenium polyhydride complexes (Section 4). This review will conclude with a summary of the thermodynamic parameters for the observed dynamic processes of rhenium polyhydride complexes (Section 6), a map of the chemical properties for the various hydride ligands in rhenium(V) polyhydride complexes (Section 7), and a brief discussion of some of the practical aspects related to these dynamic processes (Section 8). The descriptions of the known dynamic processes of rhenium polyhydride complexes are found in Section 5.

## 2. Catalytic Transformations with Rhenium Polyhydride Precatalysts

Rhenium polyhydride complexes catalyze a variety of chemical transformations. Most often, the transformation of the organic substrate(s) involves the addition or removal of hydrogen atoms from the substrate(s) during the course of the reaction. Rhenium polyhydride complexes, as precatalysts, have been shown to activate carbon-hydrogen bonds at either sp^2^ or sp^3^ carbon centers. The putative 16-electron and coordinatively unsaturated intermediate ReH_5_P_2_ (where P = a tertiary phosphine center) is thought to play a role in many catalytic transformations that occur using rhenium polyhydride complexes [2].

### 2.1. Catalytic Hydrogenation and Hydrogen Transfer Reactions

**Scheme 1:**
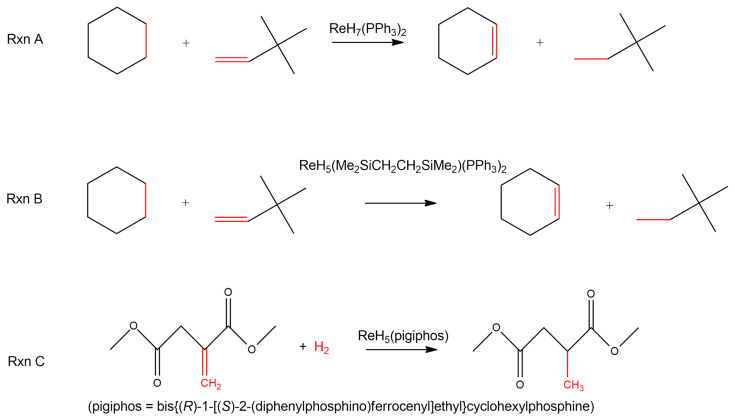
Examples of hydrogenation reactions catalyzed by rhenium polyhydride precatalysts. Complete details on all precatalysts, substrates, reaction products, and reaction conditions are available in the referenced articles: Rxn A [38], Rxn B [39], and Rxn C [40].

Catalytic transfer hydrogenation was first reported in 1983 by Felkin and coworkers. Three different rhenium(VII) polyhydride complexes (ReH_7_(PPh_3_)_2_, ReH_7_[P(*p*-fluorophenyl)_3_]_2_, or ReH_7_[P(*p*-tolyl)_3_]_2_) were found to serve as precatalysts for the conversion of cycloalkanes with six, seven, or eight carbon atoms into the corresponding cycloalkenes. 3,3-Dimethylbutene served as the hydrogen acceptor in the hydrogen transfer reactions (Scheme 1) [38]. A second example of catalytic transfer hydrogenation was reported by Crabtree and coworkers [39]. In the second report, rhenium(VII) pentahydride complexes supported by a chelating disilyl ligand (ReH_5_[1,2-bis(dimethylsilyl)benzene](PPh_3_)_2_ or ReH_5_[1,2-bis(dimethylsilyl)ethane](PPh_3_)_2_) served as precatalysts for the transfer hydrogenation of 3,3-dimethylbutene, with, again, cycloalkanes serving as the source of dihydrogen (Scheme 1) [39].

Subsequent to the reports of transfer hydrogenation using rhenium polyhydride precatalysts, simple hydrogenation using dihydrogen was also reported with a rhenium polyhydride precatalyst [40]. A prochiral olefin substrate, dimethyl itaconate, was used together with a chiral rhenium precatalyst, ReH_5_(pigiphos) (pigiphos = bis{(*R*)-1-[(*S*)-2-(diphenylphosphino)ferrocenyl]ethyl}cyclohexylphosphine), for the reported hydrogenation reaction [40]. The reduction product formed as a racemic mixture. The chiral rhenium precatalyst imparts no enantiomeric selectivity into the reduced product of the reaction. The lack of enantiomeric selectivity may be due to the dynamic nature of all eight rhenium-bound atoms in typical rheniu(V) pentahydride complexes.

### 2.2. Catalytic Carbon-Hydrogen Bond Activation Where the Hydrogen Atoms Lie Alpha to a Carbonyl Group

**Scheme 2:**
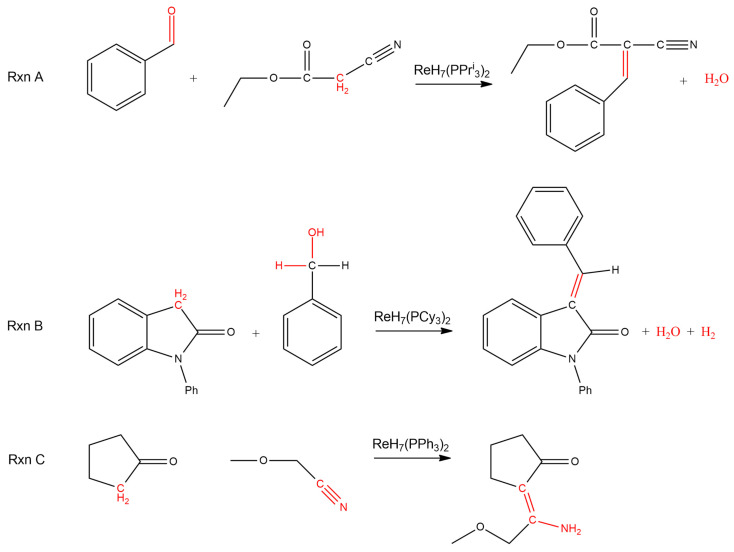
Examples of reactions catalyzed by rhenium polyhydride precatalysts in which hydrogen atoms alpha to an organic carbonyl are activated by the catalyst. Complete details on all precatalysts, substrates, reaction products, and reaction conditions are available in the referenced articles: Rxn A [41], Rxn B [42], and Rxn C [43].

Rhenium(VII) heptahydride complexes supported by tertiary phosphine ligands serve as precatalysts for several reactions that activate hydrogen atoms on carbon centers that are alpha to an organic carbonyl (Scheme 2) [41,42,43]. For the first example reaction in Scheme 2 [41], benzaldehyde reacts with ethyl 2-cyanoacetate in a condensation reaction to produce an alkene, which is conjugated with the nitrile and the carbonyl groups. Water is the second product of the reaction. The complex ReH_7_(PPr^i^_3_)_2_ serves as the precatalyst.

A second pair of reactions that activate hydrogen atoms that are alpha to organic carbonyl groups are more complex. The products of both reactions contain alkenes, which include the alpha carbon atom. The alkenes in the products are conjugated with the organic carbonyl group that was present in the original substrate for the reaction. One example reaction in Scheme 2 is a condensation reaction, which produces both water and dihydrogen [42]. No sacrificial alkene is needed for this dihydrogen-producing reaction to proceed. The precatalyst for this reaction is ReH_7_(PCy_3_)_2_.

The third example reaction in Scheme 2 of an activation of hydrogen atoms that are alpha to an organic carbonyl group starts with reactants with a total of three degrees of unsaturation (the ketone and a nitrile) and produces a single product with only two degrees of unsaturation (the ketone and an alkene) [43]. The decrease in unsaturation results from the formation of a carbon-carbon bond between the two substrates. Free dihydrogen is not formed in this reaction. The two hydrogen atoms of the carbon alpha to the carbonyl are ultimately transferred to the nitrile functional group in the product as the nitrile is reduced to an enamine, which is conjugated with the carbonyl group. The complex ReH_7_(PPh_3_)_2_ serves as the precatalyst for this catalytic transformation.

### 2.3. Amide, Ester, or Secondary Amine Formation Catalyzed by Rhenium Polyhydride Precatalysts

**Scheme 3:**
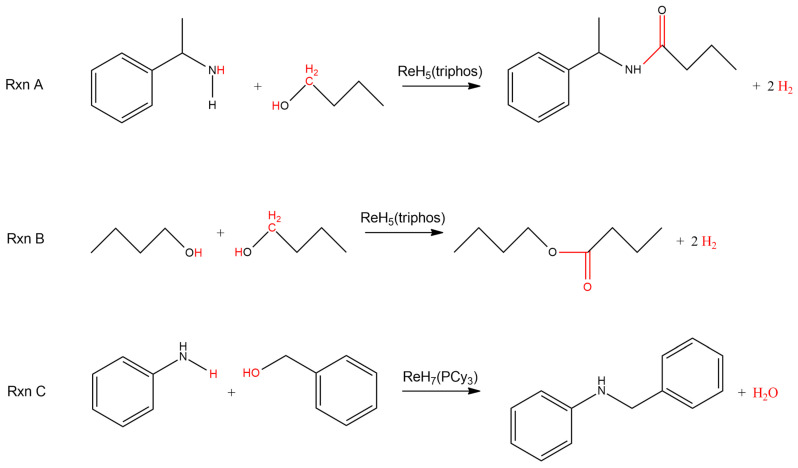
Examples of reactions in which an alcohol or an alcohol and an amine serve as the substrates for a rhenium polyhydride precatalyst. Complete details on all precatalysts, substrates, reaction products, and reaction conditions are available in the referenced articles: Rxn A and Rxn B [44], Rxn C [45].

Scheme 3 provides examples of condensation reactions that are catalyzed by rhenium polyhydride complexes. The first two reactions listed in Scheme 3 are examples of acceptorless dehydrogenation reactions as well [44]. Reaction 1 includes an alcohol and an amine reacting together to form an amide along with two dihydrogen molecules. Similarly, when the substrate is a single alcohol, the reaction proceeds to an ester and two dihydrogen molecules. The precatalyst for either the amide or ester formation is the rhenium(V) complex ReH_5_(triphos) (triphos = CH_3_C(CH_2_PPh_2_)_3_). When a primary alcohol is reacted with a primary amine in the presence of the rhenium(VII) precatalyst ReH_7_(PCy_3_)_2_, a secondary amine and water are produced in a condensation reaction (Scheme 3) [45]. The difference in the products between reactions exemplified by the first reaction in Scheme 2, a reaction between an alcohol and an amine that produces an amide, and reactions exemplified by the third reaction in Scheme 2, a reaction also between an alcohol and an amine that produces a secondary amine, presumably arises from the choice of alcohol substrate. The alcohol substrates used in [44] were entirely primary aliphatic alcohols while the alcohols used in [45] were entirely benzylic alcohols. The difference in the reaction course is presumably related to the presence or absence of protons on the carbon adjacent to the carbon of the alcohol functional group.

### 2.4. Miscellaneous Reactions Catalyzed by Rhenium Polyhydride Precatalysts

**Scheme 4:**
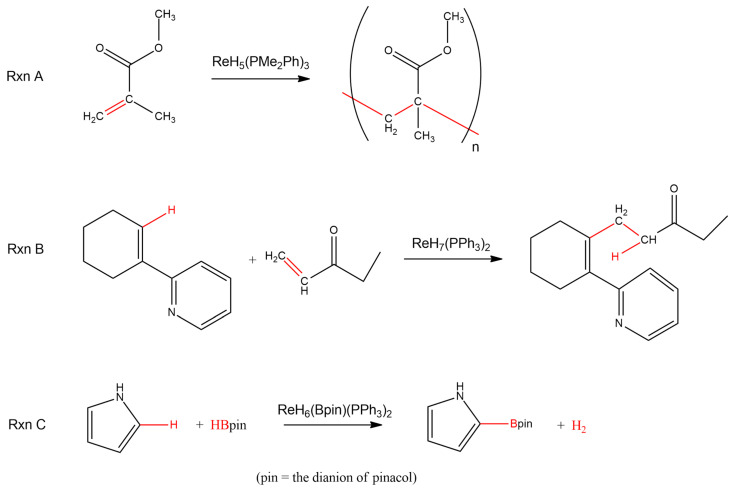
A collection of three transformations (polymerization, olefin insertion, and borylation of an aromatic heterocycle from top to bottom) that are catalyzed by rhenium polyhydride precatalysts. Each transformation has only one report in which rhenium polyhydride precatalysts are shown to accomplish such a transformation. Complete details on all precatalysts, substrates, reaction products, and reaction conditions are available in the referenced articles: Rxn A [46], Rxn B [47], and Rxn C [48].

Scheme 4 describes three singular examples of rhenium polyhydride complexes serving as precatalysts for the transformations of organic molecules. The examples in Scheme 4 likely indicate that similar reactions can be catalyzed by other rhenium polyhydride complexes as well. Reaction A in Scheme 4 is the only example of catalytic polymerization that has been facilitated by rhenium polyhydride complexes [46]. Carbon-carbon double bonds are often found in substrates and in products from other rhenium polyhydride catalyzed reactions, but this is the sole report of polymerization of olefins by rhenium polyhydride complexes. Interestingly, the rhenium(V) pentahydride complex with monodentate tertiary phophine ligands, ReH_5_(PMe_2_Ph)_3_, is an effective polymerization catalyst for methyl methacrylate while a complex with a tridentate phosphine ligand, ReH_5_(triphos), is not effective as a polymerization catalyst. This observation may indicate that loss of a phosphine ligand from the rhenium coordination sphere is an important step in activating the precatalyst ReH_5_(PMe_2_Ph)_3_.

The second reaction in Scheme 4 is an insertion reaction in which an olefin is inserted into a carbon-hydrogen bond at an sp^2^ carbon center [47]. The effect of the insertion reaction is to convert the alpha, beta unsaturated system into a saturated system and to join the beta carbon of the initially unsaturated reactant to a second unsaturated system. The reaction proceeds to couple two separate substrate molecules or to complete a ring on certain substrates that include two appropriate olefins. Carbon monoxide serves as a co-catalyst in the reaction along with the precatalyst ReH_7_(PPh_3_)_2_. The reported insertion reactions seem to be the only examples of rhenium polyhydride complexes serving as precatalysts for insertion reactions.

The final reaction in Scheme 4 is a recent observation of the catalytic activation of carbon-hydrogen bonds in heterocyclic aromatic substrates [48]. The precatalyst for the reaction, ReH_6_(Bpin)(dppe)] (pin = the dianion of pinacol), is a rare example of a rhenium center stabilized by a direct bond between boron and rhenium. The reaction replaces a hydrogen of a heteroarene such as thiophen or indole with the Bpin boryl group. The reaction is the first example for the use of a high valent rhenium complex as a borylation catalyst.

## 3. Lowest-Energy Structures of Rhenium Polyhydride Complexes

### 3.1. Nine-Coordinate Rhenium(VII) Complexes

**Figure 4:**
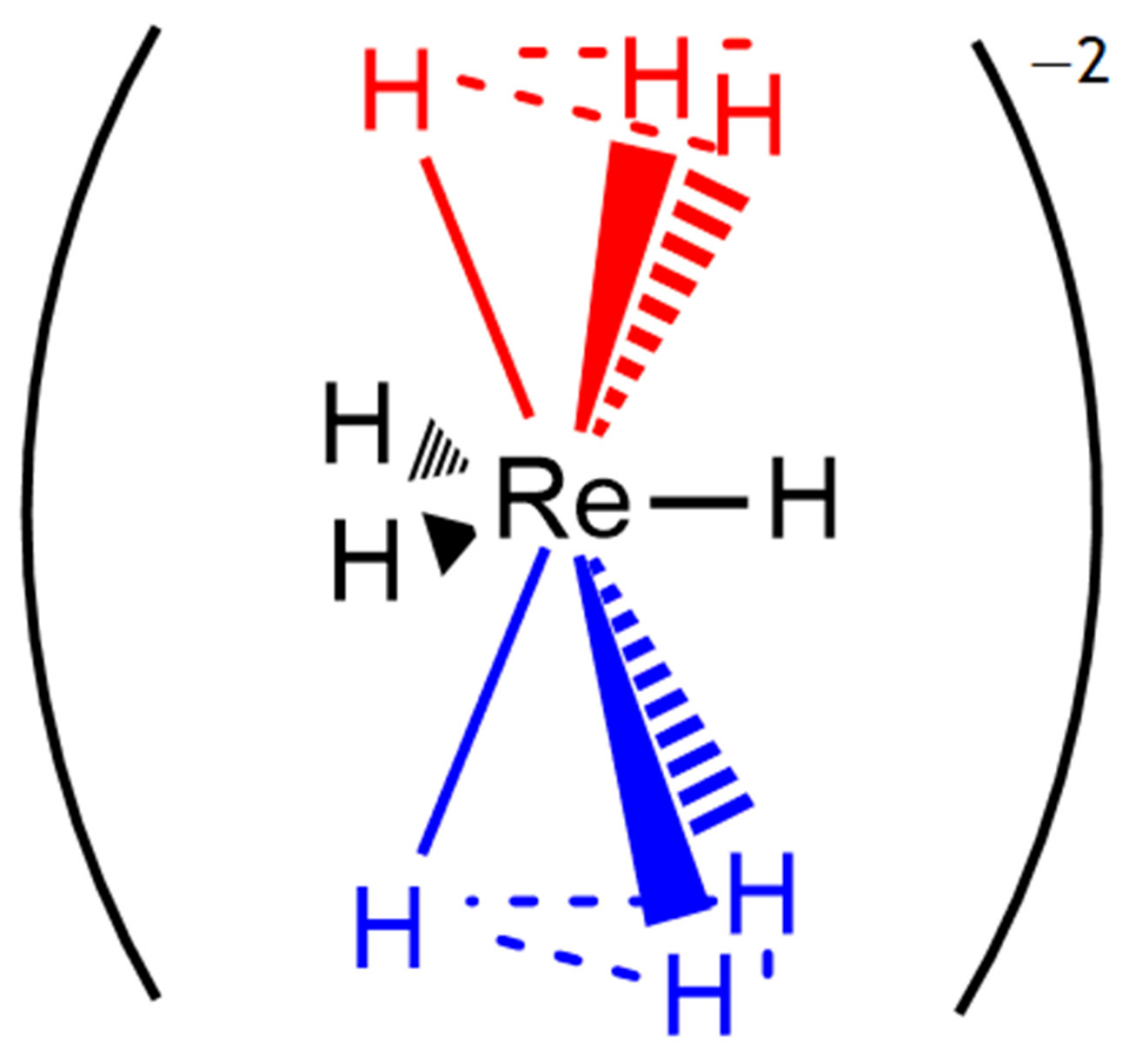
A representation of the tricapped trigonal prismatic structure of the (ReH_9_)^2−^ dianion as determined by neutron diffraction analysis [5]. The red and blue hydride ligands represent the top and bottom trigonal faces, respectively. The face-capping hydride ligands are depicted in black.

**Figure 5:**
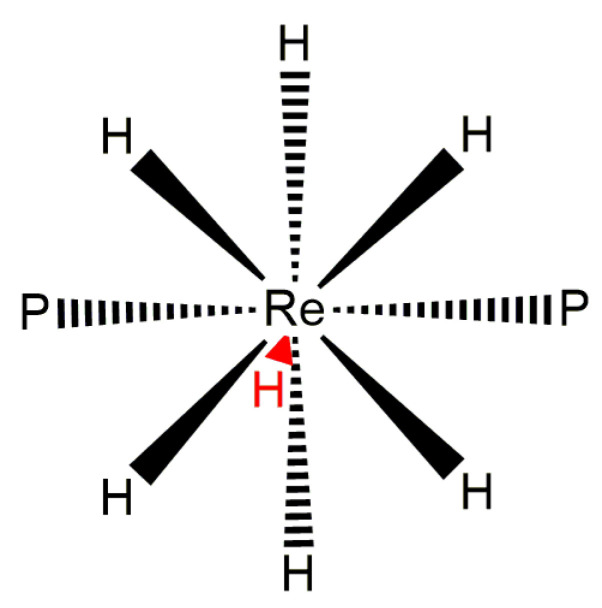
A representation of the C_2v_ capped square antiprismatic atom arrangement around rhenium in complexes of the form ReH_7_P_2_, where P = ½ dppe or P(o-tol)_3_ [13,50]. The face-capping hydride ligand is shown in red for clarity.

The complex dianion (ReH_9_)^2−^ adopts a D_3h_ tricapped trigonal prismatic geometry (Figure 4) in the solid state [5]. The complexes ReH_7_[P(*o*-tol)_3_]_2_ and ReH_7_(dppe) (dppe = 1,2-bis(diphenylphosphino)ethane) adopt a monocapped square antiprismatic geometry with a hydride ligand capping the square face of four hydride ligands and both of the tertiary phosphine ligands adopting the opposite corners of the second square face of the square antiprism (Figure 5) [13,50]. These three complexes illustrate the two common solid-state geometries that are found for rhenium(VII) polyhydride complexes: a capped square antiprism or a tricapped trigonal prism. A recent report of calculations by Kraka and coworkers found a very low energy barrier (~0.3 kcal/mol) for the transition of the (ReH_9_)^2−^ ion from its lower-energy D_3h_ tricapped trigonal prismatic geometry to a higher-energy C_2v_ monocapped square antiprism geometry [36]. Such a rearrangement between the D_3h_ and C_2v_ geometries (or vice versa) may occur in solution for many rhenium(VII) polyhydride complexes and may contribute to the general difficulty in slowing the dynamic processes of such rhenium(VII) complexes sufficiently to allow for the measurement of slow-exchange NMR spectra for such samples in solution.

### 3.2. Eight-Coordinate Rhenium(V) Complexes

**Figure 6:**
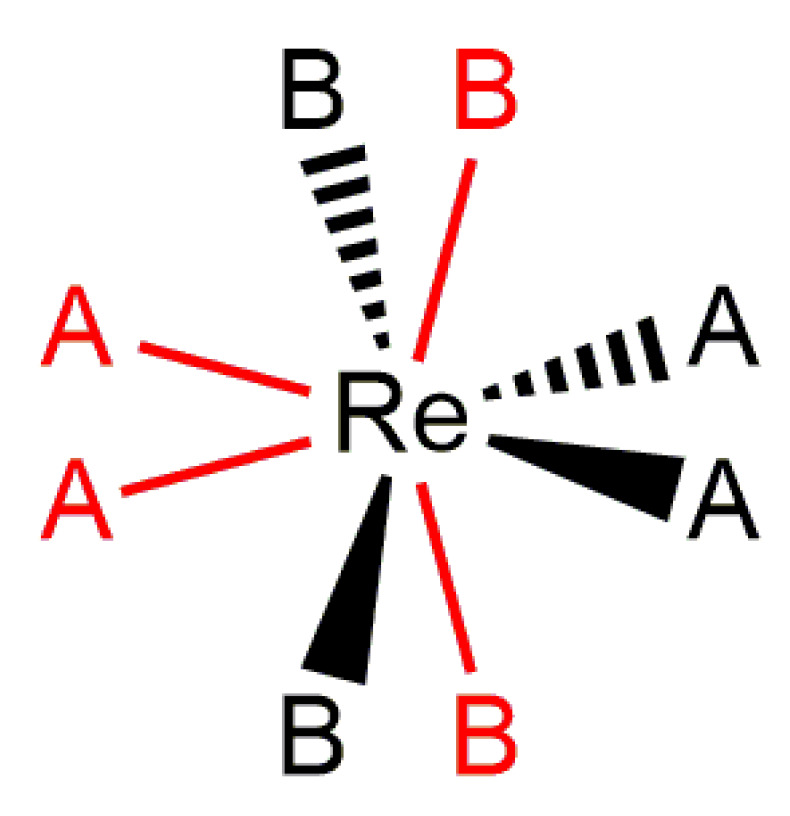
A representation of a dodecahedral arrangement of ligands around a rhenium center. The A and B coordination sites are labeled accordingly. The bonds and sites labeled in red represent a vertical trapezoid of ligands around rhenium while the bonds and sites labeled in black represent a horizontal trapezoid of ligands.

Structurally characterized eight-coordinate rhenium(V) polyhydride complexes such as ReH_5_(PMePh_2_)_3_ or [ReH_4_(PMe_2_Ph)_4_]^+^ adopt a distorted pseudododecahedral coordination geometry in the solid state (Figure 6) [51,52]. Figure 6 represents the common features of dodecahedral geometry about a rhenium(V) center. The geometry consists of two orthogonal trapezoids, with the long edges of the trapezoids arranged on opposite sides of the metal center. The coordination sites on the long edges of the trapezoids are considered as B sites and are less sterically demanding. As a general rule, bulky neutral two-electron donor ligands such as tertiary phosphines and amines adopt B coordination sites in eight-coordinate rhenium (V) complexes. When two identical tertiary phosphines are present in the coordination sphere, these phosphine ligands adopt the edge of a single trapezoid unless there is an interaction between the complex and an ionic metal center [53,54]. The more sterically demanding A sites located on the short edges of the trapezoid are occupied by hydride ligands unless a monoanionic bidentate ligand occupies a single A coordination site on the short edge of a trapezoid [3,55,56,57].

### 3.3. Seven-Coordinate Rhenium(III) Complexes

**Figure 7:**
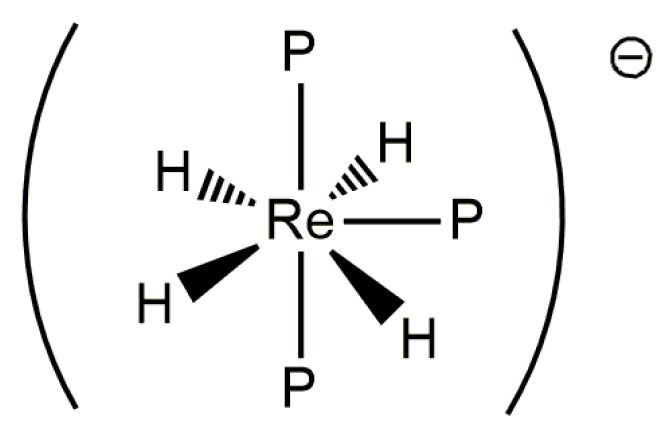
A representation of the atom arrangement for rhenium(III) tetrahydride anionic complexes [30,53].

There is limited information with regards to rhenium(III) polyhydride complexes. Only a few examples of such complexes have been prepared through the reaction of rhenium(V) pentahydride tris-phosphine complexes with KH or a similar reagent in solution [30,53]. The reaction of rhenium(V) polyhydride complexes with KH can sometimes lead to deprotonation of the rhenium(V) pentahydride centers, leaving a rhenium(III) tetrahydride anion stabilized by three tertiary phosphine centers. The structure of such anions is pentagonal bipyramidal, with both axial coordination sites occupied by tertiary phosphine ligands (Figure 7). The room-temperature ^31^P-{^1^H} NMR spectra of such complexes indicate that the phosphorus atoms exchange with one another in solution to produce a sharp singlet resonance. The mechanism of the phosphorus atom exchange has not been described.

## 4. Characterization of Dynamic Processes at Rhenium Polyhydride Complexes

Line shape fitting of dynamic NMR spectra [10,23,31,33,34,37,58,59], molecular modeling [22,27,35,36], and isotopic substitution [1,4,31,33,60,61] are the most commonly used techniques to examine the dynamic properties of rhenium polyhydride complexes. Longitudinal ^1^H NMR relaxation measurements (T_1_) of hydride ligand relaxations have been frequently used to explore the possibility of dihydrogen ligand coordination, but the technique has been shown to have issues that make it less than ideal [11,14,16,58,62]. The value of ^1^J_HD_ is generally considered to be a more reliable indicator of the presence or absence of a dihydrogen ligand by ^1^H NMR spectroscopy [17,63]. Two-dimensional ^1^H EXSY or ^1^H ROESY measurements have also been used to examine atom exchanges [23,33]. Isotope substitution has been frequently used to examine intermolecular exchange of protons or deuterons into or away from the coordination sphere of such complexes but generally not in a quantitative fashion.

### Aspects of Line Shape Fitting of Dynamic NMR Spectra of Rhenium Polyhydride Complexes

**Figure 8:**
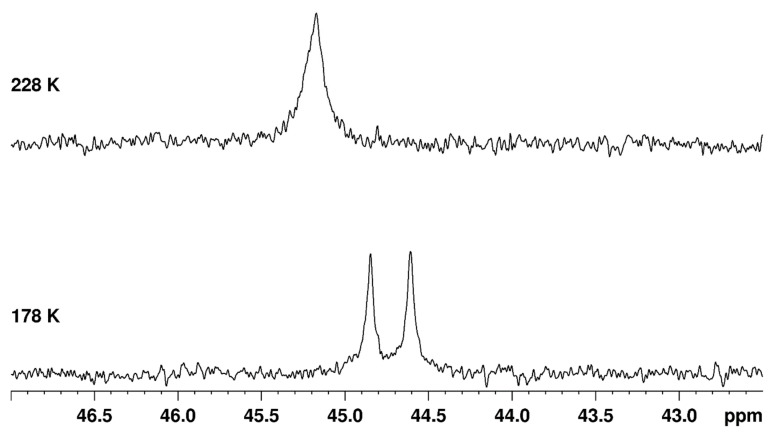
The ^31^P-{^1^H} NMR spectra of a CS_2_:d_6_-acetone (9:1) solution in the slow-exchange temperature domain (178 K) and at the coalescence temperature (228 K) for the complex ReH_5_(PPh_3_)_2_(4-phenylpyrimidine) [32].

**Figure 9:**
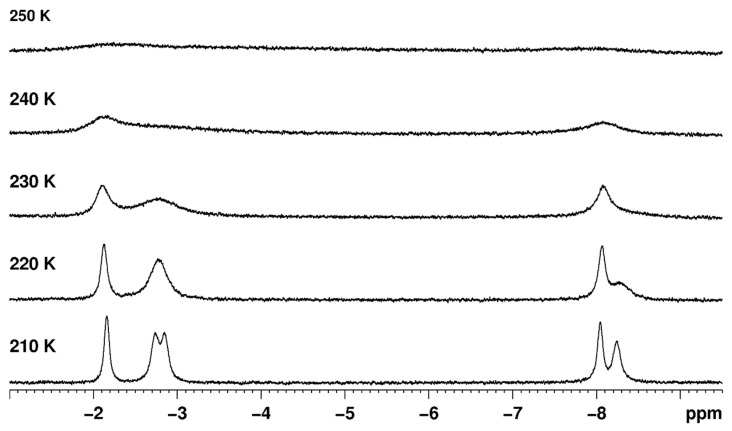
The hydride region ^1^H-{^31^P} NMR spectra of a d_8_-toluene solution of ReH_5_(PPh_3_)_2_(2-amino-3-methylbutane) measured at several temperatures. The spectrum measured at 210 K represents a slow-exchange spectrum while the remaining four spectra represent intermediate-exchange spectra [37].

As the most common experimental technique for observing the dynamic processes of rhenium polyhydride complexes, it is worthwhile to include a few aspects of line shape fitting for dynamic NMR spectra of rhenium polyhydride complexes. Variation of the hydride region of ^1^H NMR spectra as the temperature of a solution of rhenium polyhydride complexes was changed was noticed in early studies of such complexes [49]. Line shape fitting of such variation is one of the techniques used, when the slow-exchange domain of the NMR spectra can be reached, to identify the dynamic processes of rhenium polyhydride complexes and to quantify the thermodynamic parameters for such processes. An alternative NMR method of quantifying the free energy of activation for a dynamic process in which an exchange occurs between two nuclides with an equal population uses the frequency difference between the two resonances and the coalescence temperature to estimate the energy barrier at the coalescence temperature (ΔG^‡^) for the process (Figure 8) [37]. When such an exchange occurs between two resonances of unequal populations, as occurs when isomers are present [32], an estimate of the two populations, the frequency difference between the resonances, and the coalescence temperature can produce an estimate of the energy barrier for conversion of each isomer into the other isomer [64]. Either the simple two-site exchange described above or more complex situations that involve several nuclei participating in multiple confounded exchanges can also be quantified by line shape fitting of dynamic NMR spectra. The dynamic NMR temperature range must include the slow-exchange temperature domain and at least the intermediate-exchange temperature range (Figure 9).

**Figure 10:**
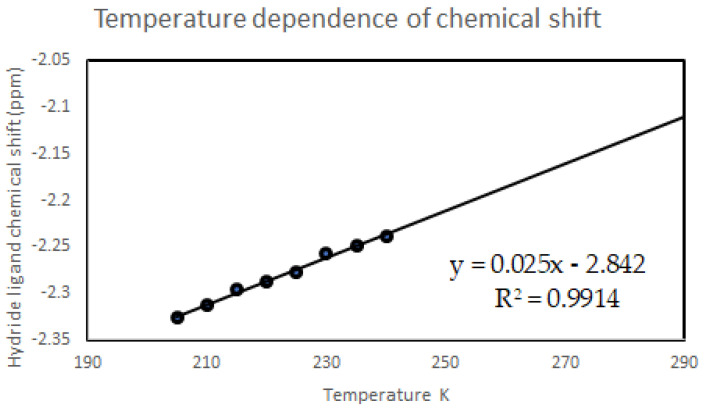
The temperature dependence of one A site hydride ligand in the complex ReH_5_(PPh_3_)_2_(*sec*-butyl amine) [65].

**Figure 11:**
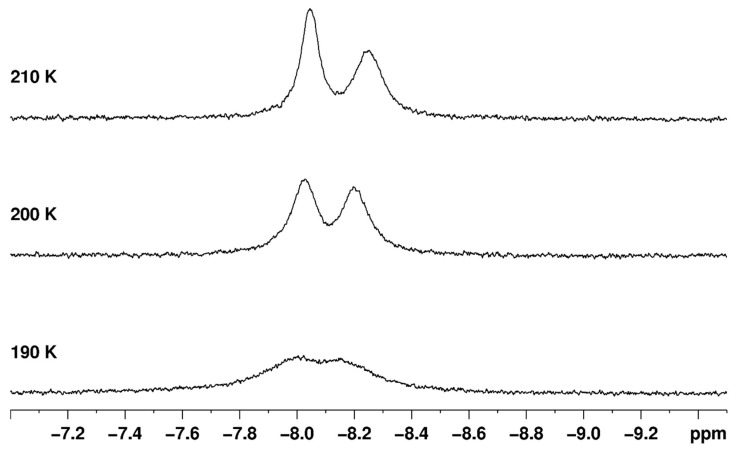
Two ^1^H-{^31^P} NMR hydride resonances for the complex ReH_5_(PPh_3_)_2_(2-amino-3-methylbutane) in a d_8_-toluene solution measured at three temperatures. The broadening of the resonances that is apparent at lower temperatures is due to precipitation of the analyte and the increased viscosity of the solvent rather than due to a dynamic exchange process.

**Figure 12:**
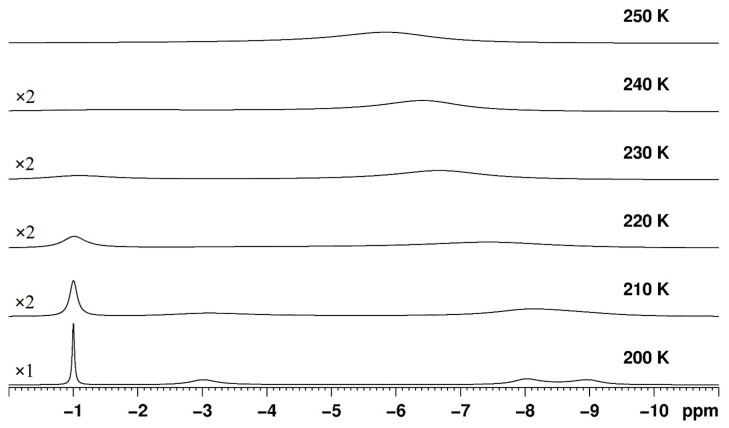
Simulations of ^1^H NMR spectra for an imagined rhenium tetrahydride complex with hydride resonances that occur at −1, −3, −8, and −9 ppm measured on a 400 MHz spectrometer at several temperatures. The three upfield resonances participate in a turnstile exchange with an energy barrier of 9 kcal/mol. The upfield and the downfield resonance participate in a direct exchange with an energy barrier of 10 kcal/mol.

When the line shape fitting technique is used to determine thermodynamic parameters for the dynamic process(es) of a rhenium polyhydride complex, or even when only the dynamic NMR spectra are presented without thermodynamic parameter determinations, a few items should be considered. First, the chemical shift of a resonance is usually temperature dependent so the temperature dependence should be determined from several slow-exchange spectra and the extrapolated chemical shifts should be used in intermediate-exchange and fast-exchange temperature domains rather than treating chemical shifts as a variable to be iteratively optimized (Figure 10). Second, at low temperatures near the freezing point of the solvent, resonances will naturally broaden due to decreased tumbling of the complex in the viscous solvent (Figure 11). Broadened resonances from changes in solvent viscosity should not be included in estimations of the rate constants for Eyring equation determinations because the additional resonance broadening overestimates the rate constants for exchange at these temperatures. Third, the appearance of any particular NMR spectrum for a dynamic chemical system is a function of the frequency difference between peaks (which depends on the combination of the chemical shift of the nuclides and the strength of the applied magnetic field) and the rate constants for each of the dynamic processes that occur. Coalescence events for multinuclei dynamic systems that participate in multiple exchange processes are simply artifacts of rate constants and frequency differences between resonances rather than markers of a particular dynamic event. Figure 12 shows a simulation of the hydride region of an ^1^H NMR spectrum, over the temperature range of 200 to 250 K for four hydride ligands that participate in only two exchange events: (1) a turnstile exchange of the three upfield hydride ligands with an energy barrier of 9 kcal/mol and (2) a pairwise exchange of the downfield and upfield hydride ligands with an energy barrier of 10 kcal/mol. It should be noted that three coalescence events are produced by only two exchange processes. It should also be noted that even though the two exchange events have different energy barriers, both exchange processes occur simultaneously, albeit at different rates, rather than sequentially from a lower energy barrier to a higher energy barrier.

## 5. Dynamic Properties That Occur at Rhenium Polyhydride Complexes

A great number of rhenium polyhydride complexes have been reported in the literature. The vast majority of rhenium polyhydride complexes exhibit one or more dynamic process(es) in the NMR spectra of a room-temperature solution. Two factors contribute to the dynamic properties of such complexes: (1) such complexes all have coordination numbers (7, 8, or 9) that intrinsically provide lower energy barriers to polytopal rearrangement than the more common coordination numbers of 4 or 6, and (2) the presence of multiple hydride ligands provides dynamic pathways for intra- and intermolecular rearrangements that are unavailable to other ligands such as oxo, hydroxide, or halide. While the dynamic nature of rhenium polyhydride complexes makes the assignment of specific chemical properties to specific atoms and sites in such complexes challenging, the presence of hydride ligands and (usually) phosphorus atoms in the rhenium coordination sphere makes NMR spectroscopy a convenient tool to identify, and oftentimes characterize, the dynamic properties of such complexes. Many dynamic processes of rhenium polyhydride complexes have been observed and this section presents those processes in a systematic fashion.

### 5.1. Intramolecular Processes

#### 5.1.1. Dynamic Observations Associated with an Amine Ligand Bound to a Rhenium(V) Center

##### Coalescence of Pyridine Ligand Protons

**Figure 13:**
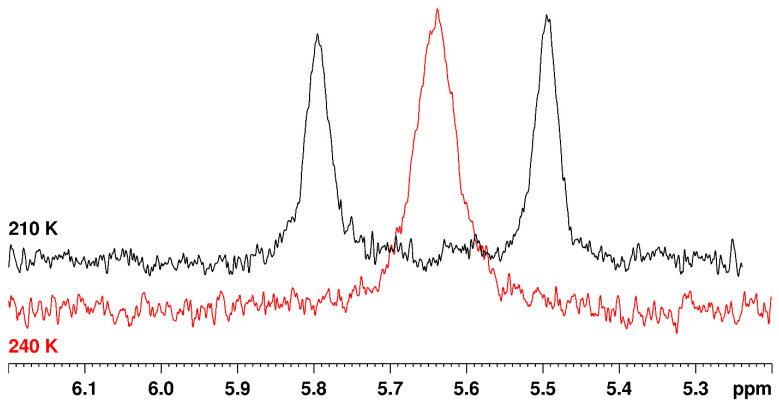
The resonances of the 3 and 5 ^1^H-{^31^P} NMR for the aromatic amine ligand of ReH_5_(PPh_3_)_2_(4-CH_3_CH(OH)py), in a d_8_-toluene solution measured in the slow-exchange domain (black trace) and at the coalescence temperature (red trace).

**Figure 14:**
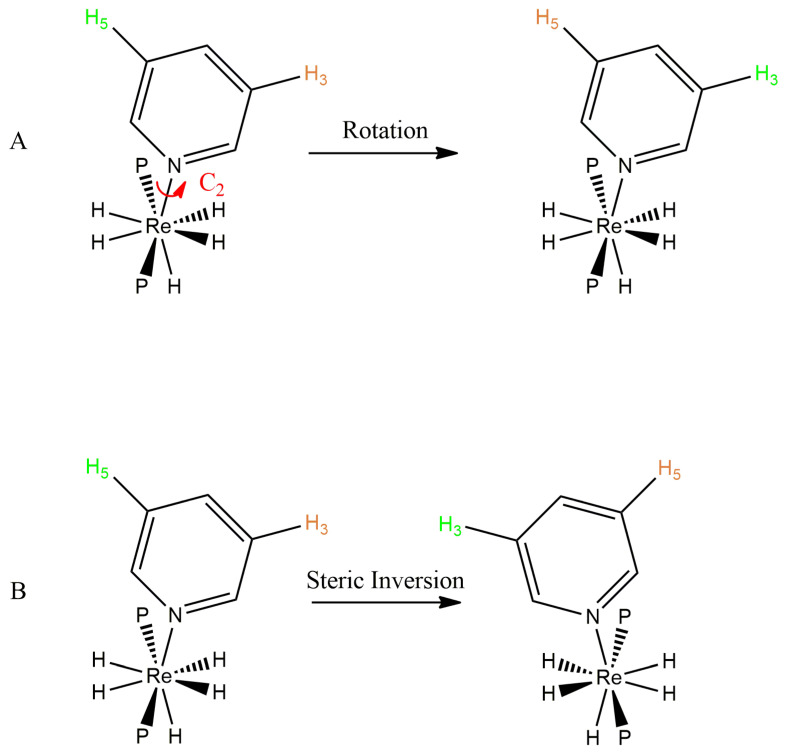
Two exchange paths that would lead to coalescence of the ^1^H NMR resonances for the 3 and 5 protons of a symmetrical aromatic amine ligand for ReH_5_(PPh_3_)_2_(amine) complexes under fast exchange conditions. Path (**A**) is a simple rotation about the nominal single bond between rhenium and nitrogen. Path (**B**) is a steric inversion that moves B site ligands across the xy plane. The steric inversion also includes an exchange of the A site hydride ligand pairs from horizontal to vertical and vice versa [32].

Dynamic ^1^H-{^31^P} NMR spectra of the complex ReH_5_(PPh_3_)_2_(py) and other symmetrically substituted aromatic amine-stabilized rhenium pentahydride complexes reveal a dynamic process involving the three and five protons of the aromatic amine ligand [23]. In the slow-exchange ^1^H NMR domain, the two protons present as unique (Figure 13). At higher temperatures, the two resonances coalesce into a single resonance through, obviously, a dynamic process involving the pyridine ligand. Figure 14 presents two potential paths by which the protons become equivalent in the fast-exchange domain ^1^H-{^31^P} NMR spectrum.

##### Interconversion of *E* and *Z* Isomers

**Figure 15:**
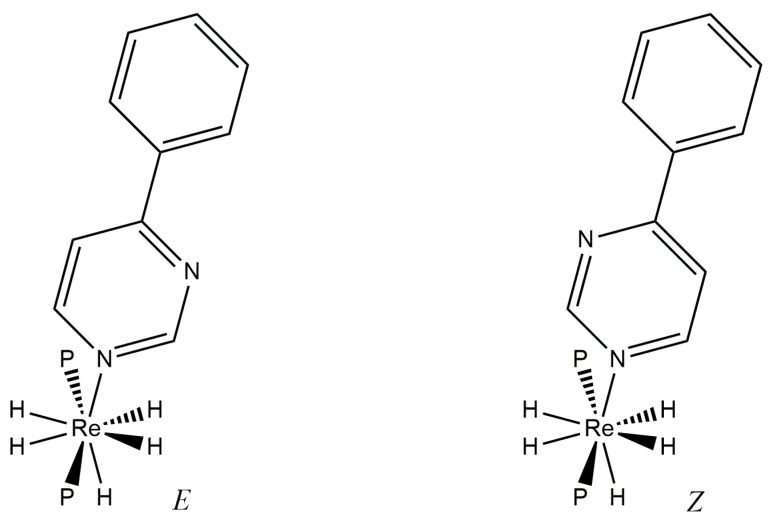
An example pair of structures that represent the *E* (**left**) and *Z* (**right**) isomers for the complex ReH_5_(PPh_3_)_2_(4-phenylpyrimidine) [32].

As would be expected given the observations of the unique 3 and 5 proton ^1^H-{^31^P} NMR resonances of a pyridine ligand on the complex ReH_5_(PPh_3_)_2_(py) (Coalescence of Pyridine Ligand Protons), unsymmetrical substitution on any aromatic amine ligand on analogous aromatic amine-stabilized complexes results in *E* and *Z* isomers of the complex (Figure 15) [32,33]. Resonances for the different isomers can be observed in the slow-exchange domain for both the ^1^H-{^31^P} and the ^31^P-{^1^H} NMR spectra of such complexes. When the unsymmetrical substituent occurs in the 2 position of the aromatic ring, the populations of the two isomers differ markedly [32]. In the fast-exchange domain, individual resonances for either isomer coalesce into a single resonance due to, presumably, the same dynamic process that occurs for ReH_5_(PPh_3_)_2_(py).

##### Coalescence of ^31^P-{^1^H} Resonances for Diastereotopic Phosphorus Centers

**Figure 16:**
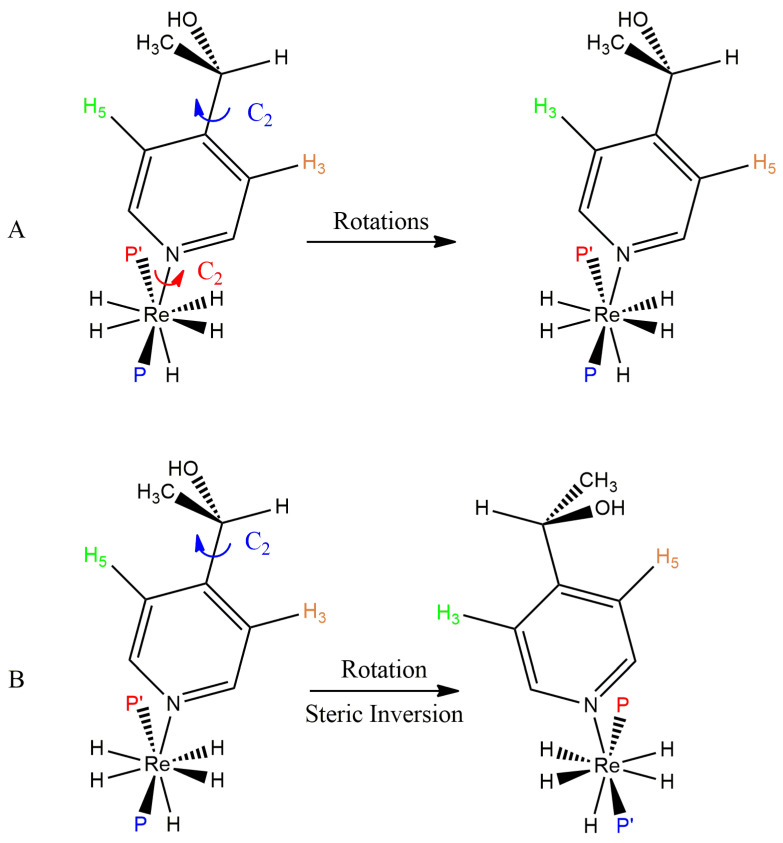
Two paths that lead to coalescence of the ^1^H NMR resonances for the 3 and 5 protons of symmetrically substituted aromatic amine complexes of the form ReH_5_(PPh_3_)_2_(amine). Path (**A**) leads only to coalescence of the 3 and 5 aromatic amine proton resonances in the ^1^H NMR spectra. Path (**B**), the steric inversion path, leads to coalescence of ^1^H NMR 3 and 5 aromatic proton resonances and coalescence of diastereotopic phosphorus ^31^P NMR resonances under fast exchange conditions [37].

The inclusion of a chiral substituent in the 4 position of a pyridine ligand or inclusion of a chiral aliphatic amine into complexes of the form ReH_5_(PPh_3_)_2_(amine) produces two doublet resonances for diastereotopic phosphorus atoms in the slow-exchange domain of the ^31^P-{^1^H} NMR spectra for some such complexes [37,65]. In the fast-exchange domain of the ^31^P-{^1^H} NMR spectra of such complexes, the pair of doublets coalesce into a sharp singlet through, presumably, the same dynamic process that occurs when the amine ligand is pyridine. For the complex with a chiral substituent in the 4 position of pyridine, ReH_5_(PPh_3_)_2_[4-CH_3_CH(OH)py], the 3 and 5 protons of the substituted pyridine ligand present as unique in the slow-exchange ^1^H NMR spectrum of the complex and these unique resonances coalesce into a sharp singlet in the fast-exchange domain ^1^H NMR spectrum of the complex (Figure 13). Figure 16 shows that fast rotation about the Re-N bond in the complex ReH_5_(PPh_3_)_2_[4-CH_3_CH(OH)py] will make the 3 and 5 ^1^H NMR resonances equivalent in the fast-exchange spectrum but will not affect the coalescence of the ^31^P-{^1^H} diastereotopic phosphorus atom resonances. For the complex of interest, the energy barriers for the exchange of 3 and 5 protons or for the coalescence of the diastereotopic phosphorus atom resonances are essentially the same [37]. Of the two choices depicted in Figure 16, the results of the investigation of the complex ReH_5_(PPh_3_)_2_[4-CH_3_CH(OH)py] support a mechanism in which the 3 and 5 proton of aromatic amine ligands and the interconversion of *E* and *Z* isomers occurs through a process that inverts the steric perspective of the two phosphorus atoms with respect to the nitrogen atom found in a B site on the second trapezoid.

#### 5.1.2. Dynamic Observations Associated with Phosphorus Atoms

##### Dynamic Observations Associated with Three Phosphorus Atoms That Participate in Intramolecular Exchange

Aside from the dynamic observations associated with diastereotopic phosphorus atoms, nearly all rhenium polyhydride complexes that include identical phosphorus atoms, whether bound to rhenium(V) or rhenium(VII), present as equivalent on the NMR timescale at room temperature [1,2,34,51,59,62,66]. Most of the complexes with three identical phosphorus atoms are found with rhenium(V) centers. The complexes with three equivalent phosphorus atoms bound to rhenium(VII) are typically prepared by protonation of neutral rhenium pentahydride complexes, supported by three tertiary phosphine centers, to form material that can be formulated as (ReH_6_P_3_)^+^ (P = tertiary phosphine centers) (although some of these protonation products can be better formulated with a dihydrogen ligand as [ReH_4_P_3_(H_2_)]^+^ [8,58,66]). The complexes of rhenium(III) with three identical phosphorus atoms are formed by deprotonation of neutral rhenium pentahydride complexes supported by three tertiary phosphine centers [30,53]. The phosphorus atoms, in such complexes, occur as either three monodentate tertiary phosphine centers such as ReH_5_(PPh_3_)_3_ or as three tertiary phosphine centers in a tridentate ligand such as ReH_5_(triphos) [1,67]. A variety of supporting ligand sets can be found for such complexes: four or five or six hydride ligands [1,2,8,30,52,53,54,62,66]; four hydride ligands and an anion such as iodide [8,34,66]; or four hydride ligands and a neutral ligand such as a nitrile, an isocyanide, or another tertiary phosphine center [8,34,52,59,66,68]. With an exception for complexes that form tautomers [7,9], any such complex that has been examined in the slow-exchange ^31^P-{^1^H} NMR domain presents as two equivalent phosphorus atoms and one unique phosphorus atom, oftentimes with significant coupling shown for the two resonances, which coalesce to a single resonance in the fast-exchange NMR domain.

**Figure 17:**
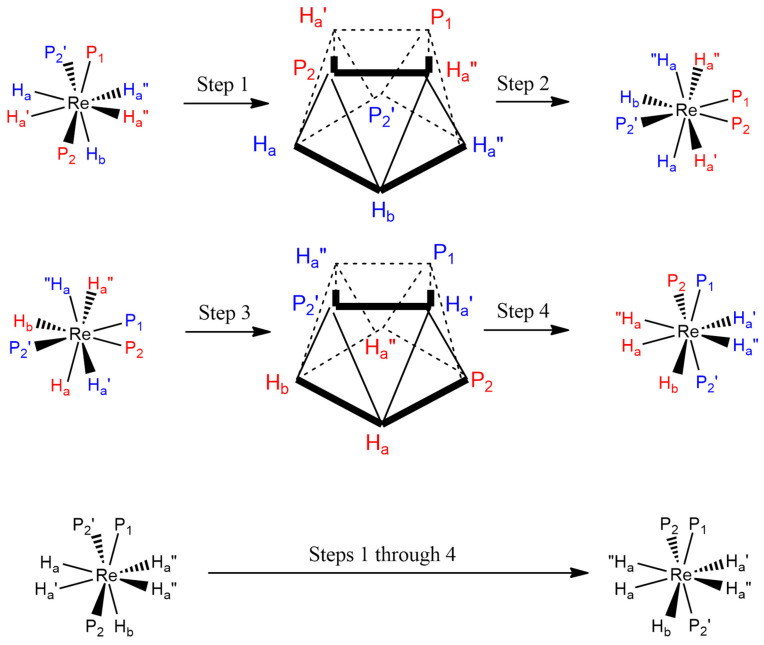
A path that leads to synchronous exchange of B site phosphorus atoms and A site hydride ligands. The red and blue colors for Steps 1 and 2 or Steps 3 and 4 indicate diamond arrangements of ligands, which transition to square faces of the square pyramidal state. Steps 1 and 2 combined move A site ligands in the initial structure into B sites in the final structure and vice versa. Step 3 begins with the inverted ligand states that resulted from Step 2. The lower representation demonstrates that such a path, under fast exchange conditions, will lead to coalescence of all ^31^P or ^1^H hydride resonances under fast-exchange NMR conditions [33,34].

Line shape fitting of dynamic ^31^P-{^1^H} NMR spectra cannot differentiate the mechanism for the dynamic process with respect to whether the process occurs as a pairwise exchange or occurs in a turnstile exchange. The process that exchanges three identical phosphorus atoms bound to eight-coordinate rhenium(V) centers, however, is not the process depicted in Figure 14B. The depicted process, which only changes the steric perspective between the atoms on the long edges of the two trapezoids, would never make an equivalent pair of phosphorus atoms and a unique phosphorus atom equivalent on the NMR timescale. For the eight-coordinate rhenium(V) complexes with identical phosphorus atoms, the dodecahedron to square antiprism to dodecahedron rearrangement described for true dodecahedral complexes seems likely to also describe the rearrangement of phosphorus atoms in the rhenium(V) complexes mentioned in this section (Figure 17) [33,34]. Such a rearrangement also fits with observations of hydride ligand rearrangements (see Pairwise Exchange of Four A Site Hydride Ligands at Rhenium(V) Centersbelow).

##### Dynamic ^31^P-{^1^H} Observations That Occur for Certain Rhenium(V) Complexes Ascribed to Tautomers

**Figure 18:**
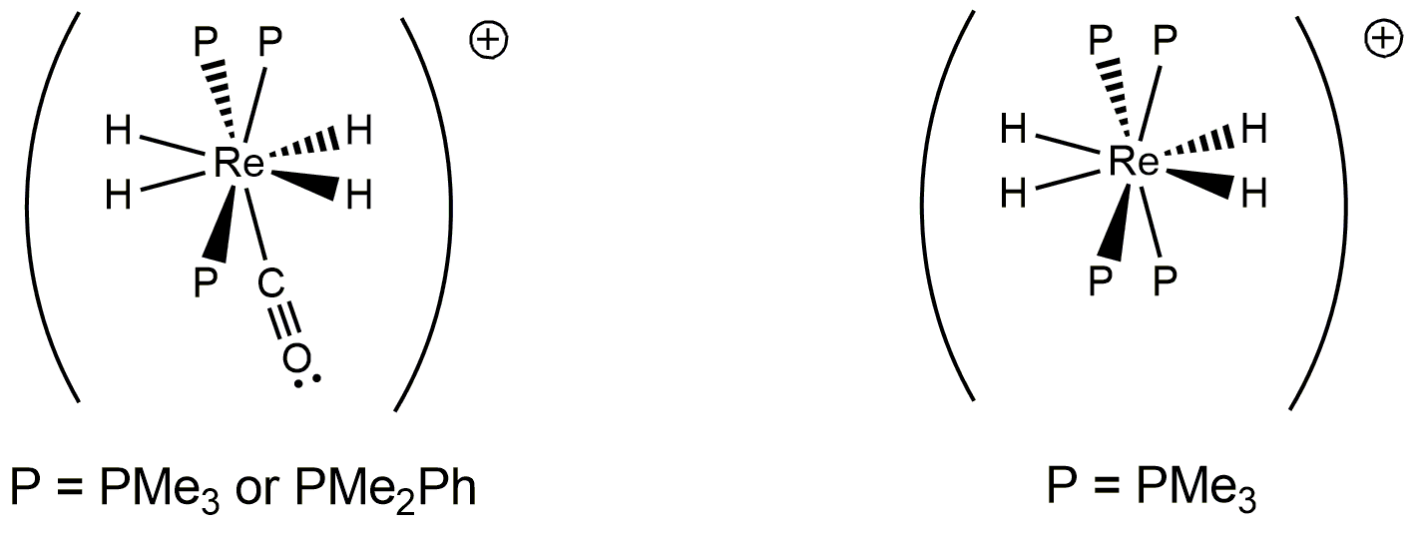
Representations of the structures of a pair of complexes, which occur as tautomers in solution (**left**) or produce anomalous NMR resonances (**right**) [7,9,68].

The three cations [ReH_4_(PMe_3_)_4_]^+^, [ReH_4_(PMe_3_)_3_(CO)]^+^, and [ReH_4_(PMe_2_Ph)_3_(CO)]^+^ present NMR characteristics that differ from other rhenium(V) polyhydride complexes stabilized by three tertiary phosphine centers [7,9,68]. At room temperature, the complex [ReH_4_(PMe_3_)_4_]^+^ exhibits a broad singlet phosphorus resonance that sharpens as the temperature is increased [68]. Furthermore, at lower temperatures, the complex [ReH_4_(PMe_2_Ph)_3_(CO)]^+^ is characterized by two pairs of ^31^P-{^1^H} NMR resonances, with each pair of resonances occurring at a ratio of 2:1 [9]. Neither of these observations are consistent with the predicted structures of each complex as presented in Figure 18. Wilkinson’s tetrakistrimethylphosphine cation, on the right in Figure 18, should present a sharp ^31^P-{^1^H} NMR singlet at all temperatures since all four phosphorus atoms would be chemically equivalent by that measurement. Berke’s [ReH_4_(PMe_2_Ph)_3_(CO)]^+^ complex should present only two ^31^P-{^1^H} NMR resonances in the slow exchange domain. Both Berke and Crabtree, who studied the [ReH_4_P_3_(CO)]^+^ (P = PMe_3_ or PMe_2_Ph) complexes, interpreted their results as evidence of tautomers with the formulas [ReH_4_P_3_(CO)]^+^ and [ReH_2_P_3_(CO)(H_2_)]^+^. Wilkinson’s complex, which was prepared and characterized before Kubas’ first report of a dihydrogen ligand, presents ^31^P-{^1^H} NMR evidence that is akin to the observations by Berke and by Crabtree. Unfortunately, no complex from this intriguing series of three complexes has been characterized by diffraction methods, although Caulton and coworkers characterized the complex [ReH_4_(PMe_2_Ph)_4_]^+^ by neutron diffraction [52].

##### Lack of Observations of Phosphorus Atom Rearrangements for Rhenium(VII) Complexes

Aside from the rhenium(VII) complexes of the form [ReH_6_P_3_]^+^ (P = tertiary phosphine or 1/3 tridentate phosphine) mentioned in Dynamic Observations Associated with Three Phosphorus Atoms that Participate in Intramolecular Exchange, no physical study has directly addressed heavy atom rearrangement at rhenium(VII) polyhydride complexes. The ^31^P-{^1^H} NMR resonances of such complexes present as sharp singlets at all temperatures [6,10]. Given recent results in a molecular modeling paper by Kraka and coworkers [36], it seems likely that such complexes undergo a pseudorotational exchange between monocapped square antiprism geometries and tricapped trigonal prismatic geometries. Preparation of a rhenium(VII) polyhydride complex that includes two identical phosphorus atoms and a ligand that includes a chiral center seems to be the most likely path to test whether two phosphorus centers in such complexes participate in dynamic rearrangements.

##### Observation of ^31^P NMR Resonances from Diastereotopic Phosphorus Atoms in a Room-Temperature Solution of a Rhenium(V) Tetrahydride Complex

**Figure 19:**
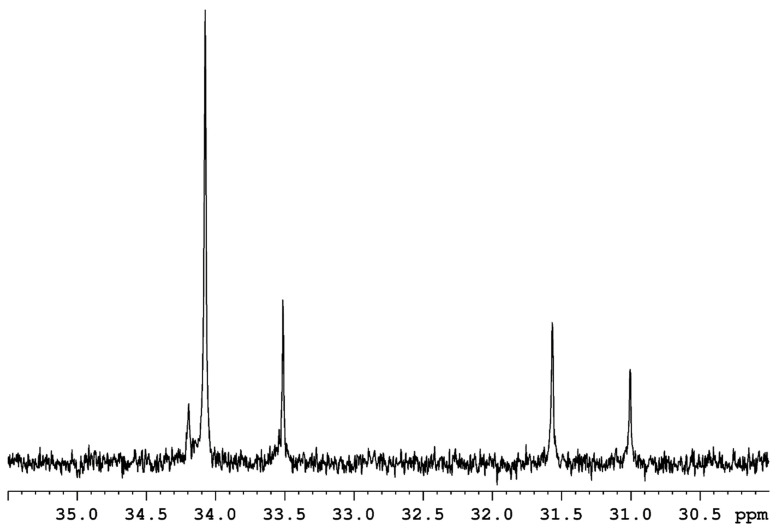
A room-temperature ^31^P-{^1^H} NMR spectrum of a d_8_-toluene solution for the complex ReH_4_[1,2-(C_6_H_4_)CH=NCH(Me)Ph](PPh_3_)_2_. The resonance at 34.1 ppm is due to a significant impurity.

Preliminary work from our group suggests that a comparatively rigid bidentate ligand, an *ortho*-metallated benzaldimine ligand, which includes a chiral center, arrests the phosphorus atom rearrangement that is observed in most other rhenium(V) polyhydride complexes. Figure 19 presents the ^31^P-{^1^H} NMR resonances of the complex ReH_4_[η^2^-(1,2-C_6_H_4_)CH=NCH(Me)Ph] along with a significant impurity. The coupled pair of ^31^P-{^1^H} doublets remains unchanged to temperatures of 340 K, at which point the complex begins to decompose. This result suggests that the inclusion of a relatively rigid chelating ligand into the coordination sphere of a rhenium polyhydride complex may provide a means to stop the dynamic rearrangement of the heavy rhenium-bound atoms. The chemical properties of specific atoms at specific coordination sites should be more easily examined in solution if the pseudorotation or pseudorotation-like heavy atom rearrangement can, in fact, be arrested.

### 5.2. Dynamic Processes Associated with Hydride Ligands

The so-called fluxional behavior of rhenium polyhydride complexes, in solution at room temperature, is a descriptor that does a disservice to our understanding of the dynamic processes that occur for such complexes. Rather than the hydride ligands existing in an unknowable state of flux, the hydride ligands appear to participate in only a handful of dynamic processes, which move hydride ligands in predictable patterns within a rhenium coordination sphere and, in many cases, easily exchange protons that reside in the coordination sphere as hydride ligands with protons from beyond the rhenium coordination sphere. Interestingly, on occasions, the protons from beyond the rhenium coordination sphere that exchange with hydride ligands begin the exchange process bound to a carbon atom of a solute molecule. A thorough understanding of which hydride ligand sites readily exchange protons with carbon atoms and the manner(s) by which such exchange occurs would help in the rational design of rhenium polyhydride catalysts for the transformation of small organic molecules. The building of such an understanding must start with an understanding of all of the dynamic processes within a particular rhenium polyhydride complex.

The structure of this manuscript has presented the dynamic behavior of heavy atoms bound to rhenium before addressing the dynamic behavior of hydride ligands for two reasons. First, heavy atoms appear to participate in only a single psuedorotation or pseudorotation-like rearrangement, so the dynamic behavior of any heavy atom is not confounded by participating in multiple dynamic processes [33,34,35,37,59]. Therefore, a single pseudorotational or pseudorotational-like dynamic process within the complex can be readily characterized. Second, the thermodynamic characteristics of dynamic processes seem to indicate that hydride ligands participate in the same pseudorotation or pseudorotation-like dynamic process that the heavy atoms participate in [33,34,35,37,59]. Synchronicity between heavy atom rearrangement and one hydride ligand mode of rearrangement simplifies efforts to identify any remaining dynamic processes that the hydride ligands participate in. The presence of a synchronous pair of observable dynamic processes (one observed for heavy atoms, such as phosphorus, and a second observed for hydride ligands) may even provide a means to test theoretical descriptions of such rearrangements predicted by molecular modeling.

#### 5.2.1. Intramolecular Dynamic Processes of Hydride Ligands

##### Pairwise Exchange of Adjacent Hydride Ligands

**Figure 20:**
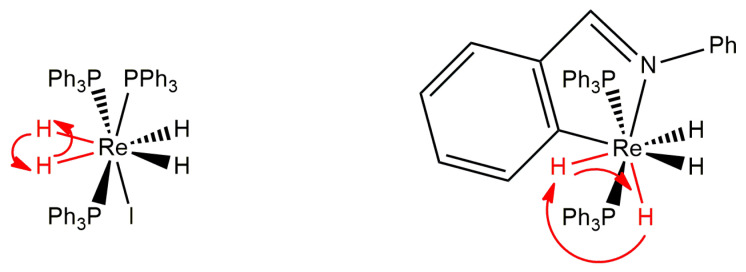
Two examples of direct exchange between two hydride ligands. The figure on the left represents the direct exchange of two inequivalent A site hydride ligands. The figure on the right represents the direct exchange of the unique B site hydride ligand with one of the two inequivalent A site hydride ligands [34].

Reports of pairwise exchange of adjacent hydride ligands are few for rhenium polyhydride complexes. Line shape analysis of dynamic ^1^H-{^31^P} NMR spectra for a series of phosphite-stabilized rhenium(V) pentahydride complexes has reported that such complexes exchange the inequivalent A site hydride ligands [10,58]. A second such exchange of inequivalent A site hydride ligands was observed by line shape analysis for two rhenium(V) tetrahydride complexes in which all of the hydride ligands occupy A sites [34]. The same report found that a pair of rhenium(V) tetrahydride complexes stabilized by a chelating ligand that occupies both coordination sites (one A and one B site) on a non-parallel edge of a trapezoid exchanges the lone B site hydride ligand with the unique A site hydride ligand in a pairwise fashion (Figure 20) [34]. This latter report is the only reported example of a B site hydride ligand exchanging directly with a single A site hydride ligand from the opposite side of the metal center.

**Figure 21:**
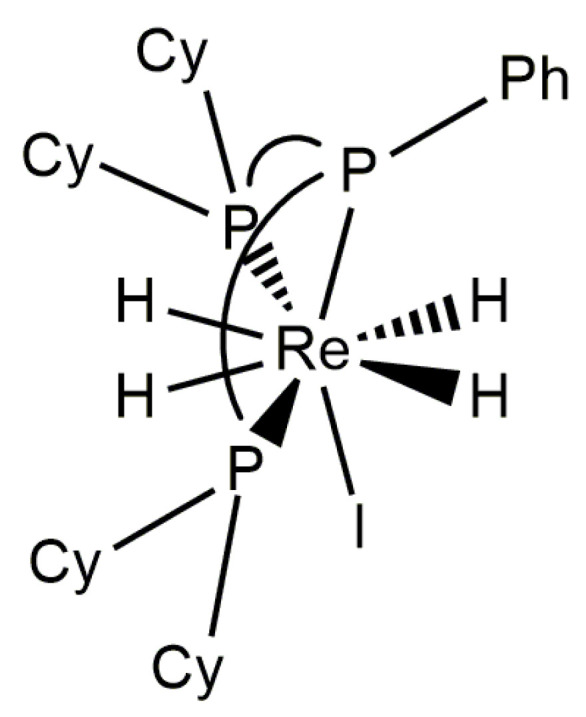
A representation of a potential structure for the complex ReH_4_I(Cyttp) [8].

In Observations of ^31^P NMR Resonances from Diastereotopic Phosphorus Atoms in a Room Temperature Solution of a Rhenium(V) Tetrahydride Complex, it was noted that a relatively rigid chelating ligand, such as an ortho-metallated benzaldimine, halts the pseudorotation or pseudorotation-like rearrangement of phosphorus atoms at rhenium(V) complexes. The bulky chelating tridentate ligand Cyttp ligand (Cyttp = PhP(CH_2_CH_2_CH_2_PCy_2_)_2_) also apparently halts any pseudorotation or pseudorotation-like rearrangement of rhenium(V) polyhydride complexes [8]. Although the dynamic ^1^H-{^31^P} NMR spectra of these complexes have not been analyzed by line shape fitting, a series of [ReH_4_(Cyttp)L]^+^ cations (L = MeCN, *t*-BuNC, CyNC, or P(OMe_3_)_3_) (Figure 21) and the neutral complex ReH_4_I(Cyttp) all exhibit three hydride resonances in room-temperature solution ^1^H-{^31^P} NMR spectra with relative intensities of 1:2:1. The downfield hydride resonance and upfield hydride resonance apparently arise from two inequivalent A site hydride ligands while the central resonance apparently arises from a pair of chemically equivalent hydride ligands. Given that the upfield and downfield hydride resonances sharpen as the temperature of the sample is decreased, it can be presumed that the change in the peak shape arises from exchange between the two unique A site hydride ligands as was seen for two similar complexes [34]. The individual ^1^H-{^31^P} NMR resonances for a pair of inequivalent exchanging hydride ligands, in room-temperature solutions, apparently is due to the large frequency difference between the exchanging hydride ligand resonances combined with a slow or nonexistent pseudorotation or pseudorotation-like rearrangement for such complexes. This series of complexes represents rare examples of rhenium polyhydride complexes in which all of the chemically distinguishable ^1^H NMR hydride resonances are apparent in room-temperature solutions of the rhenium polyhydride complex.

##### Turnstile Exchange of Three Adjacent Hydride Ligands

**Figure 22:**
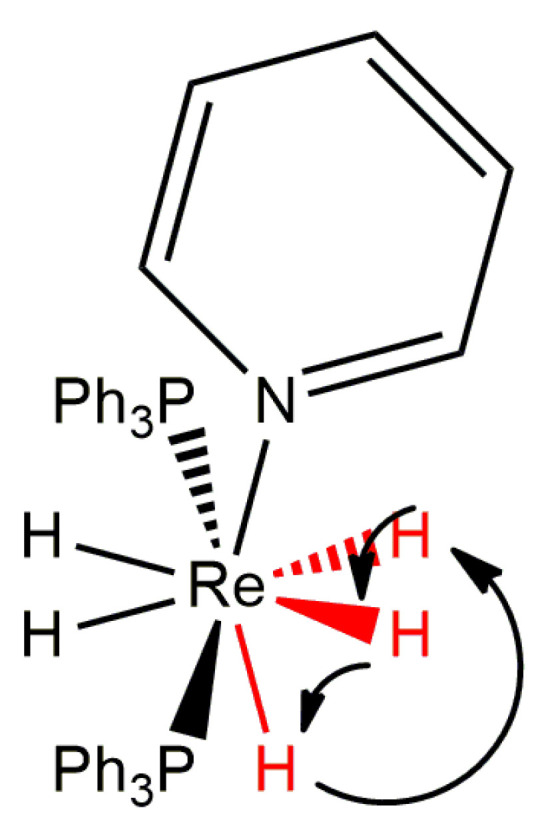
A representation of a three-arm turnstile rotation of two hydride ligands in equivalent A sites and the unique B site hydride ligand of ReH_5_(PPh_3_)_2_(py) [23].

The dynamic hydride ligand exchange processes for rhenium(V) pentahydride complexes appear to always include a turnstile exchange of three adjacent hydride ligands that reside in two equivalent A sites and an adjacent B site (Figure 22) when these features are present [10,23,27,33,34,35,37,58]. Line shape analysis of the dynamic ^1^H-{^31^P} NMR spectra of such complexes always results in a model that includes such an exchange [10,23,27,33,34,37,58]. The dynamic ^1^H-{^31^P} NMR spectra for some complexes with the same three-hydride structural feature that have not been analyzed by line shape fitting sometimes yield low-temperature hydride region spectra that include three hydride ^1^H-{^31^P} NMR resonances, which integrate as 1:1:3 [69]. The resonance, in such spectra, that integrates for three protons is likely to result from a turnstile exchange of a pair of equivalent A site hydride ligands with the unique B site hydride ligand in which the slow-exchange temperature domain has not been reached for the turnstile exchange process. The rhenium(V) tetrahydride complexes ReH_4_[η^2^-(1,2-C_6_H_4_)CH=NPh] and ReH_4_[η^2^-2-(1,2-C_6_H_4_)py] also exhibit the turnstile exchange of an equivalent pair of hydride ligands with the unique B site hydride ligand, although the thermodynamic properties of this turnstile exchange differ markedly from the parameters found for other pentahydride rhenium(V) complexes (Section 6).

A recent computational report on a pair of rhenium(V) pentahydride complexes found a mechanism by which a series of three turnstile rearrangements, with the second of the three turnstiles (the basal turnstile) incorporated into a molecular pseudorotation, describes the entire rearrangement of such complexes [35]. The reported lateral turnstile rotations are similar to a turnstile rotation reported by Crabtree and Eisenstein previously [27]. The turnstile rotations that have been reported by line shape analysis of dynamic NMR spectra, therefore, strongly agree with the theoretical calculations on such complexes. The computational support for the turnstile rearrangement is important in that the line shape fitting model cannot distinguish a turnstile exchange of three hydride ligands from an alternating pairwise exchange of the same three ligands, due to the limitations of the line shape fitting technique. Line shape fitting of dynamic NMR spectra and molecular modeling of exchange processes are complementary techniques for understanding the dynamic process of rhenium polyhydride complexes. A recent molecular modeling report regarding the ion (ReH_9_)^2−^ found a turnstile exchange for the tricapped trigonal prismatic form of the ion [36].

**Figure 23:**
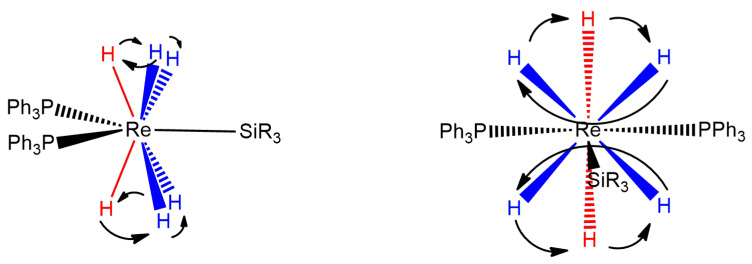
Possible structures for the complexes ReH_6_(SiR_3_)(PPh_3_)_2_ [70].

An interesting rhenium(VII) complex for which a slow-exchange ^1^H NMR spectrum of the hydride region may have been measured is ReH_6_(SiPh_3_)(PPh_3_)_2_ [70]. At 213 K, measured at 250 MHz in a solution of CD_2_Cl_2_, the room-temperature triplet hydride resonance resolves into two hydride resonances with intensities of 2:4. A 2:4 ratio of hydride ligands corresponds to the potential lowest-energy structure for either of the common nine-coordinate geometries that occur for rhenium(VII) polyhydride complexes (Figure 23). Turnstile exchanges of three adjacent hydride ligands for either of the common geometries would be the only dynamic process necessary to make all six hydride ligands equivalent in the fast-exchange domain ^1^H-{^31^P} NMR spectrum of the complex.

##### Pairwise Exchange of Four A Site Hydride Ligands at Rhenium(V) Centers

Several reports of line shape fitting of the dynamic NMR spectra of rhenium(V) tetra- or pentahydride complexes have reported an exchange of the identities of the four A site hydride ligands within these complexes [10,23,24,33,34,37,58,59]. Nearly all such complexes have a pair of equivalent hydride ligands that reside on the short edge of a trapezoid that is bisected by a plane of symmetry (Figure 22). The remaining two A site hydride ligands occupy the short edge of the second trapezoid and these two hydride ligands are chemically inequivalent. A pseudorotation or pseudorotation-like event for the molecule causes the identity of these four hydride ligands to exchange in a pairwise fashion; the symmetrical pair becomes the unsymmetrical pair and vice versa. A theoretical investigation of the rearrangements that occur for the complex ReH_5_(PPh_3_)_2_(py) suggests that the pseudorotational step in the mechanism, a basal turnstile hydride ligand exchange, is synchronous with an exchange of steric perspectives of the phosphorus atom pair with respect to the amine ligand [35]. An investigation of the dynamic processes of the small series of complexes ReH_5_(PPh_3_)_2_(amine) (amine = (*R*)-α,4-dimethylbenzylamine), (*R*)-2-amino-3-methylbutane, (*R*)-α-methyl-4-pyridinemethanol], or *sec*-butylamine) stabilized by a chiral amine ligand found that it was the pairwise exchange of A site hydride ligands that exchanged most synchronously with the exchange of the steric perspective of the diastereotopic phosphorus atoms [37,65]. The difference between the theoretical prediction and the line shape fitting of dynamic NMR spectra with respect to the key pseudorotation or pseudorotation-like step likely arises from whether the key pseudorotation or pseudorotation-like step includes an interaction with a solvent molecule or not (see Section 6).

##### Circle Dance Exchange of Five Hydride Ligands

**Figure 24:**
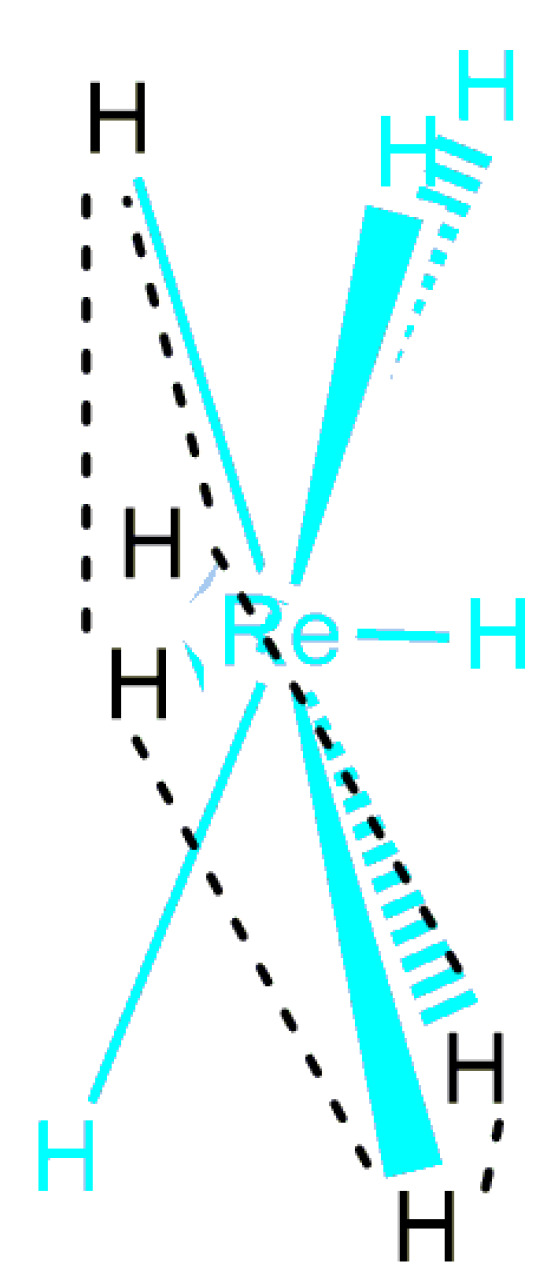
A representation of the pair of edge hydride ligands, pair of capping hydride ligands, and one last edge hydride ligand, which participate in one of six circle dance hydride ligand exchanges [36].

A recent computational paper that examined the (ReH_9_)^2−^ dianion identified an exchange that moves five hydride ligands in an orbital-like procession around the metal center (Figure 24) [36]. The calculated energy barrier to the circle dance exchange of hydride ligands is small: ~5 kcal/mol. The exchange is reported for the solid-state form of the salt K_2_ReH_9_. This circle dance exchange of hydride ligands appears to likely only occur for complexes with a tricapped trigonal prismatic geometry because all five participating hydride ligands must essentially lie on a single plane of the complex.

##### Exchange of Hydride Ligands with a Dihydrogen Ligand

**Figure 25:**
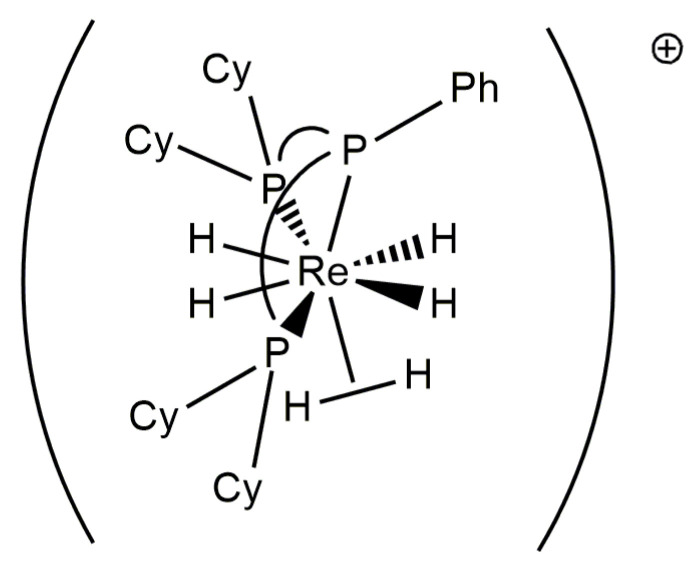
A representation of the crystal structure of the mixed hydride-dihydrogen cation [ReH_4_(Cyttp)(H_2_)]^+^ [8].

Much effort has been directed towards preparing rhenium polyhydride complexes with one or more dihydrogen ligands as part of the coordination sphere [6,7,8,9,10,69,71]. Efforts have also been directed towards the development of an unambiguous characterization for a dihydrogen ligand in the coordination sphere of rhenium polyhydride complexes [11,12,13,14,15,16]. Some of the clearest examples of success along the lines of preparing complexes that combine multiple hydride ligands and a dihydrogen ligand in the same coordination sphere are the reports of [ReH_4_(Cyttp)(H_2_)]^+^ and [ReH_6_{Cy_2_PO(CH_2_)_2_OPCy_2_}(H_2_)](BF_4_), both of which are prepared by protonation of neutral rhenium polyhydride precursors [8,10]. The clearest evidence for the formulation of [ReH_4_(Cyttp)(H_2_)]^+^ is the crystal structure, which is a clear example of a pseudododecahedral rhenium(V) cation with the dihydrogen ligand residing in a B coordination site (Figure 25) [8]. For the protonation reactions of rhenium(VII) cations such as ReH_7_{Cy_2_PO(CH_2_)_2_OPCy_2_}, it is clear that the proton must add to a hydride ligand in that rhenium(VII) is already in its group oxidation state [6,10,71]. An important aspect regarding the example complexes that contain dihydrogen as a ligand is that the protons of the dihydrogen ligand exchange rapidly, even at low temperatures on the NMR timescale, with the protons of hydride ligands. No dynamic process has been characterized for proton exchanges between hydride ligands and dihydrogen ligands for complexes that do not occur as tautomers.

##### Tautomeric Exchange of a Dihydrogen Ligand with a Pair of Hydride Ligands

Two examples have been reported of complexes that exist, in solution, as an equilibrium mixture of tautomers [7,9]. The tautomers occur from different hydrogen atom binding modes. The tautomer equilibrium exists for the monocationic complexes [ReH_4_P_3_(CO)]^+^ and [ReH_2_P_3_(CO)(H_2_)]^+^ (P = PMe_3_ or PMe_2_Ph). The tautomer mixtures are prepared from the protonation of the neutral rhenium(III) complexes ReH_3_P_3_(CO). Hall provided a theoretical analysis of the tautomer mixture [22]. While it has been shown that other rhenium polyhydride compounds, such as [ReH_4_(Cyttp)(H_2_)]^+^, are best formulated with a mix of hydrogen atom ligands (hydride and dihydrogen) in the coordination sphere, only two examples of tautomeric mixtures have been reported. Energy barriers to exchange between the two tautomeric forms have been calculated to be small: ~8 kcal/mol [22].

#### 5.2.2. Intermolecular Dynamic Exchange of Inner Sphere Hydride Ligands with Hydrogen from Beyond the Inner Coordination sphere

##### Proton Exchange between Hydride Ligands and Exchangeable Protons of Small Molecules in Solution

Chatt and Coffey first reported the ability of deuteride ligands bound to the complex ReD_7_(PPh_3_)_2_ to readily exchange with hydroxyl protons of ethanol in the presence of a drop of hydrochloric acid catalyst [1]. Similar exchanges have been observed in many subsequent reports of rhenium(VII) polyhydride complexes [3,5,8,31,60,72,73]. The exchange phenomenon, when it occurs, occurs with a variety of different hydrogen sources from the solvent system, including hydrogen bound to oxygen (as water or alcohol), nitrogen, carbon, or boron. The mechanism for the proton exchange is not well understood. The phenomenon generally proceeds faster (lower energy barrier) with more basic exchanging partners such as amines and water than with less basic exchanging partners such as hydroxyl groups or aromatic molecules [31].

**Figure 26:**
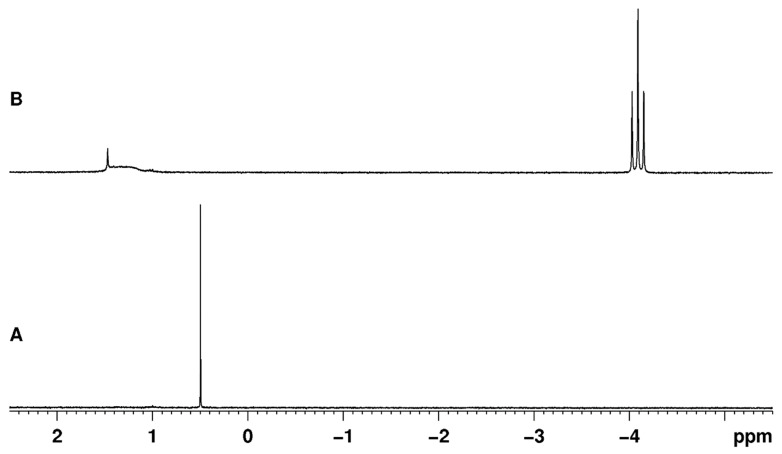
The ^1^H NMR spectrum of adventitious water in C_6_D_6_ before (**A**) and after (**B**) the addition of ReH_7_(PPh_3_)_2_ (**B**). The change in the water resonance (~1.5 ppm in Spectrum B) corresponds to a sharp resonance for the water proton that is not exchanging with the rhenium complex and a broad resonance for the associated and exchanging proton of water.

Crabtree and coworkers established that there is an attractive interaction between solutes with exchangeable protons and rhenium(VII) polyhydride complexes such as ReH_7_(dppe) [26]. Similarly, the molecule ReH_7_(PMe_3_)_2_ holds enough attraction for water that the molecular couple ReH_7_(PMe_3_)_2_^.^H_2_O has been observed in the gas phase by mass spectrometry [74]. In a similar phenomenon, when a sample of ReH_7_(PPh_3_)_2_ is added to d_8_-toluene, which contains adventitious water, a proton exchange proceeds between water protons and rhenium-bound hydride ligands [31]. Unexpectedly, in the ^1^H-{^31^P} NMR spectrum of the solution, the broadened adventitious water resonance (broadened due to proton exchange with the added rhenium complex) shifts downfield, away from the hydride resonance (its exchanging partner) rather than upfield (towards its exchanging partner) (Figure 26). The downfield shift of the adventitious water resonance in this situation is similar to the shift for the adventitious water ^1^H-{^31^P} NMR resonance that occurs upon association of water with the ReH_5_(PPh_3_)_2_(amine) series of complexes [33,37]. As with the ReH_5_(PPh_3_)_2_(amine) complexes, in the presence of ReH_7_(PPh_3_)_2_, the ^1^H NMR signal for adventitious water in a C_6_D_6_ solution shows different resonances for the two protons of water, indicating a significant attraction (and proton exchange giving rise to the broadening of one water proton) between one water proton and at least one hydride ligand of the rhenium complex. Crabtree and other workers have attributed the attractive interactions between solute molecules and rhenium polyhydride complexes to dihydrogen bonding [24,25,26,27,28,37,75,76,77]. It appears that dihydrogen bonding is a phenomenon that occurs between a variety of rhenium polyhydride complexes and solute molecules. Dihydrogen bonding is an attraction between hydrogen atoms that is necessary for proton exchange between solute molecules and hydride ligands, but for a molecule such as ReH_7_(PMe_3_)_2_, the attraction itself is not sufficient for proton exchange to occur in a rapid fashion [74].

**Figure 27:**
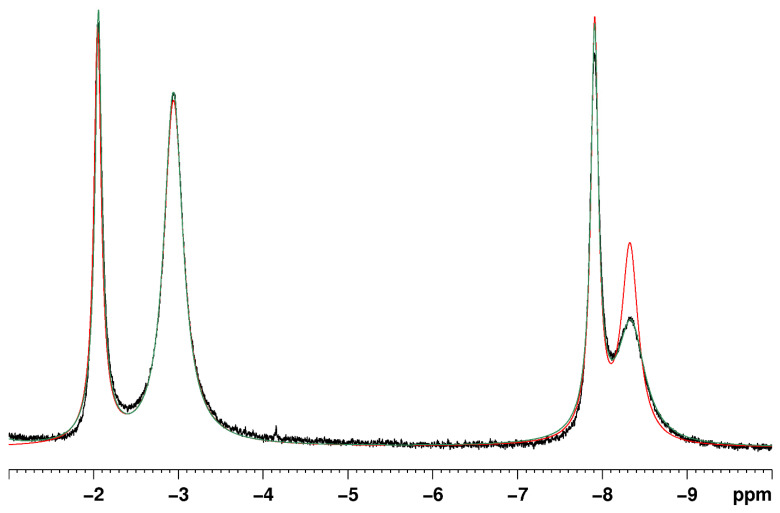
The hydride region for the ^1^H NMR spectrum of a d_8_-toluene solution of ReH_5_(PPh_3_)_2_(η^1^-ethylene diamine) at 220 K (blue trace). The green trace is a simulation of the spectrum, which includes a turnstile exchange of hydride ligands, a pairwise exchange of the A site hydride ligands, and a proton exchange between water and the B site hydride ligand, which resonates at −8.4 ppm. The red trace is the best fit for the same exchange model without the exchange of protons between water and the B site hydride ligand [33,37].

**Figure 28:**
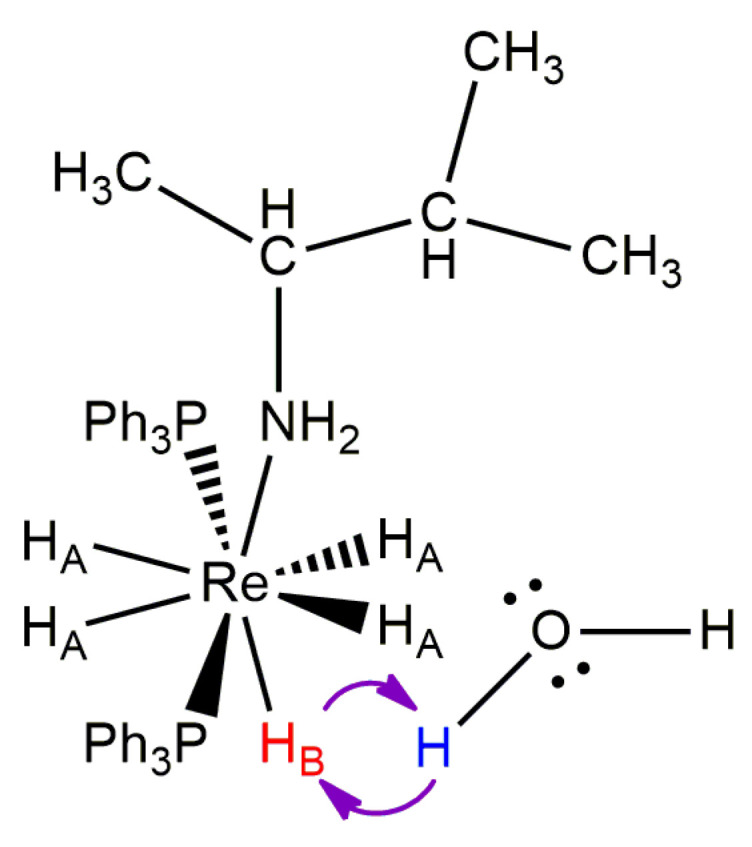
A representation of the exchange of protons between a water molecule and the B site hydride ligand of ReH_5_(PPh_3_)_2_(*sec*-butyl amine) [65].

Line shape fitting of dynamic ^1^H-{^31^P} NMR spectra for complexes of the form ReH_5_(PPh_3_)_2_(amine) indicates that the unique B site hydride ligand in such complexes participates in a proton exchange that moves the hydride ligand beyond the inner coordination sphere of rhenium (Figure 27 and Figure 28) [33,37,65]. The movement of the unique B site hydride ligand beyond the rhenium coordination sphere is seen regardless of whether the model used to fit the observed spectra is based upon Crabtree’s original report of the dynamic processes of such complexes or if the model is based upon a recent theoretical examination of these systems [27,35,37,65]. The line shape fitting results are supported by isotope exchange experiments and by observations, by ^1^H-{^31^P} NMR, of differentiated water proton resonances that arise from association and/or exchange of only one water proton with the rhenium complex of interest [33,37]. Considerable evidence supports a strong attraction between exchangeable protons and the unique B site hydride ligand of ReH_5_(PPh_3_)_2_(amine) complexes, whether in solution or in the solid state [26,37,75,77].

There is no proven mechanism for proton exchange between exchangeable protons of solute molecules and rhenium hydride ligands. For the series of ReH_5_(PPh_3_)_2_(amine) complexes, the exchanging hydride ligand has been identified as the unique B site hydride ligand. No evidence has been presented that the more common A site hydride ligands that occur in rhenium(V) polyhydride complexes participate in proton exchange with solute molecules. A B site hydride ligand in rhenium(V) polyhydride complexes is adjacent to the electron density from the rhenium lone pair of electrons while the A site hydride ligands are well removed from the lone pair electron density of rhenium. Therefore, it is interesting to note that for the rhenium(VII) polyhydride complexes, which have been reported to readily exchange protons between solute molecules and a hydride ligand [3,5,8,31,60,72,73], such complexes all include aromatic rings on an ancillary tertiary phosphine or arsine ligand. To the best of our knowledge, there have been no reports of rapid intermolecular proton exchange for rhenium(VII) polyhydride complexes stabilized solely by phosphine or arsine ligands devoid of aromatic rings [31]. An examination of what role, if any, the electron density from the ancillary ligands can play in proton exchange at rhenium(VII) polyhydride centers may produce interesting results.

##### Exchange of Hydride Ligands with Free Dihydrogen

Dihydrogen is more frequently used to probe the nature of a reaction or decomposition mechanism than as an actual source for hydrogen atom exchange with the inner coordination sphere rhenium hydride ligands. Recently, it was demonstrated that HD will exchange into the coordination sphere of rhenium(VII) polyhydride complexes stabilized by NHC ligands [4].

##### Exchange of Hydride Ligands with Hydrogen Bound to Carbon

Rhenium polyhydride complexes can transform carbon-hydrogen, boron-hydrogen, and silicon-hydrogen bonds. Frequently, the transformation of such bonds occurs in a stoichiometric fashion [1,3,4,32,39,40,55,56,59,60,70,78,79,80,81,82,83,84,85,86,87,88,89,90,91,92,93]. Stoichiometric transformations of carbon hydrogen bonds will frequently lead to rearrangement of the organic substrate and/or coordination of carbon to rhenium.

With respect to the mechanisms of carbon-hydrogen bond transformations by rhenium(VII) polyhydride complexes, rhenium(VII) complexes participate in hydrogen transfer reactions that start with alkane substrates [85]. In such transfer hydrogenation reactions, the active form of rhenium is typically a 16-electron rhenium center that has reductively lost dihydrogen [92]. Many of the other organic substrates that are transformed by rhenium polyhydride centers, such as terminal alkynes and alcohols, have some acid character in thf. Morris has reported extensively on the acid properties of metal hydride bonds in nonaqueous solvents [54,94,95]. Acid–base reactions may also be important in the activation of carbon-hydrogen bonds by rhenium polyhydride complexes, although a study was unable to observe a significant attraction between rhenium(V) polyhydride complexes and terminal alkyne protons in solution [28].

##### Direct Exchange of Hydride Ligands between the Inner Coordination Spheres of Two Complexes

Two reports describe the transfer of hydride ligands directly from one metal complex to another metal complex [31,96]. Both reports involve the complex ReH_7_(PPh_3_)_2_ or its reactive form ReH_5_(PPh_3_)_2_, which is likely to be formed by the loss of dihydrogen from the ReH_7_(PPh_3_)_2_ complex, as the receiving end of the hydride ligand transfer. In one report, ReH_7_(PPh_3_)_2_ serves as a precatalyst for the catalytic dehydrogenation of the complex ReH_3_(PPh_3_)_2_(C_5_H_6_) to form the dehydrogenated product ReH_2_(C_p_)(PPh_3_)_2_. In the second report, an in situ mixture of H and D isotopomers of ReH_(7–x)_D_x_(PPh_3_)_2_ (x = 0, 1, 2, 3, 4, 5, 6, 7) (which is formed slowly from ReH_7_(PPh_3_)_2_ dissolved in 9:1 C_6_D_6_:CD_3_OD) combines with fresh ReH_7_(PPh_3_)_2_, in the same solution, to immediately redistribute the relative concentrations of the various isoopomers (i.e., ReH_7_(PPh_3_)_2_, ReH_6_D(PPh_3_)_2_, ReH_5_D_2_(PPh_3_)_2_, …) to correspond to the statistically predicted concentrations of each isotopomer. The statistically predicted concentrations of each isotopomer are based on the new ratio of H and D within the rhenium inner coordination spheres following the addition of the fresh ReH_7_(PPh_3_)_2_ into the system.

## 6. Thermodynamic Characterizations of Dynamic Processes

### 6.1. Exchange of Protons between Water and a Unique B Site Hydride Ligand

**Figure 29:**
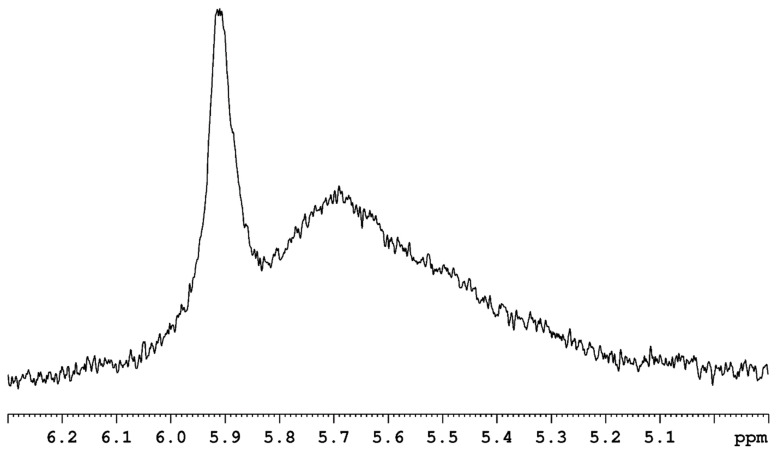
The ^1^H-{^31^P} NMR signal for adventitious water in a d_8_-toluene solution of ReH_5_(PPh_3_)_2_(2-amino-3-methylbutane).

Three reports of line shape fitting for dynamic ^1^H-{^31^P} NMR spectra indicate that proton exchange occurs between adventitious or intentional water and the unique B site hydride ligand of complexes with the form ReH_5_(PPh_3_)_2_(amine) [33,37,65]. The line shape fitting observations are supported by ^1^H-{^31^P} two-dimensional exchange spectroscopy observations [33], an isotopic substitution experiment [33], and observation of differentiated water protons in the dynamic ^1^H-{^31^P} NMR spectra of such complexes (Figure 29) [37]. The line shape fitting results provide estimates for ΔH^‡^ for the proton exchange that range from 11.0 to 20.7 kcal/mol, with a mean of 13.4 kcal/mol and a standard deviation of 3.9 (Table 1). The estimates for ΔS^‡^, for the same process, range from 4.4 to 47.3 cal/mol∙K, with a mean of 10.6 cal/mol∙K and a standard deviation of 5.6 (Table 1). Estimates of ΔG^‡^_260_ for the proton exchange range from 8.4 to 10.5 cal/mol∙K, with a mean of 9.6 cal/mol∙K and a standard deviation of 0.7 (Table 1). The individual characteristics of each complex, and the average characteristics, are consistent with a dissociative process. The dissociative characteristics of the activation parameters indicate that a solute molecule such as water associates with either the unique B site hydride ligand or the nearby rhenium lone pair of electrons. Crabtree and coworkers demonstrated such an association in both the solid state and in solution [26,29,75,77]. At some point during that association, the bond between oxygen and the proton belonging to water is cleaved and a new bond forms between oxygen and the unique hydride ligand at the B coordination site. Dissociation of the newly formed water molecule that includes the proton of the former B site hydride ligand constitutes the highest energy step in the proton exchange process.

**Figure 30:**
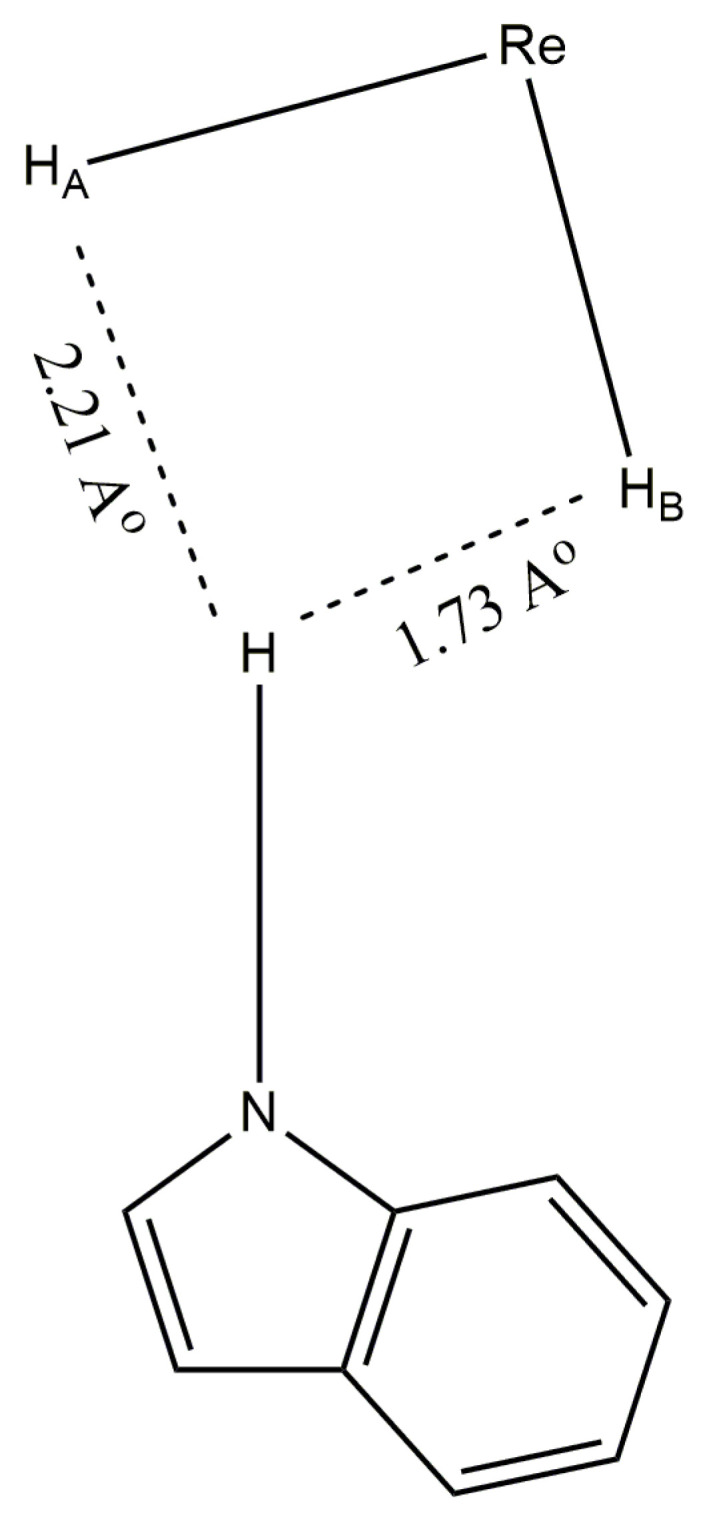
A representation of the solid-state interaction between the N-bound proton of indole and two hydride ligands (one A site and one B site) for the complex ReH_5_(PPh_3_)_3_ [29].

Two structural characterization reports by Crabtree and coworkers found exchangeable protons bound to nitrogen (indole in one example and imidazole in the second example) that exhibit short hydrogen–hydrogen contact distances, in the solid state, with rhenium-bound hydride ligands [29,75]. The shortest hydrogen–hydrogen distance, in both examples, is the distance between the nitrogen-bound proton and the unique B site hydride ligand of the rhenium(V) pentahydride complex. The nitrogen-bound proton also has a short contact with the nearest A site hydride ligand from the side of rhenium that is opposite the B site hydride ligand (Figure 30). The nitrogen-bound proton, by virtue of its closer contact with the B site hydride ligand, lies essentially on the xy plane that would be defined for a true dodecahedral complex. It should be noted that the rhenium lone pair of electrons occupies the dxy orbital of rhenium. Thus, the nitrogen-bound proton, in both of Crabtree’s structurally characterized complexes, situates itself not only near the B site hydride ligand (the hydride ligand that exchanges with a water proton for similar complexes in solution) but also situates itself near the rhenium lone pair of electrons. With respect to the exchange of protons between water and the unique B site hydride ligand of ReH_5_(PPh_3_)_2_(amine) complexes, the rhenium lone pair of electrons, a lone pair of electrons on oxygen, and bonding pairs of electrons between O and H as well as Re and H, combine with the two protons that exchange to result in a proton exchange in a dissociative fashion.

### 6.2. Psuedorotation or Pseudorotation-like Rearrangement of Rhenium Polyhydride Complexes

A lack of pseudorotation or pseudorotation-like behavior appears to be the exception for the heavy atoms bound to rhenium polyhydride complexes. Complexes with three identical phosphorus atoms, two identical phosphorus atoms and a chiral center, or even just an aromatic amine ligand at an eight-coordinate center all exhibit motions, by NMR spectroscopy of room-temperature solutions, that are consistent with pseudorotation-like behavior (Section 5.1) with only two exceptions due to chelating ligands. The rhenium polyhydride complexes in which the activation parameters for pseudorotation-like motions have been characterized by line shape fitting of dynamic NMR spectra are collected into Table 2 [10,33,34,37,58,59,65]. All of the rhenium polyhydride complexes in Table 2 are eight-coordinate rhenium(V) complexes. Some complexes in Table 2 include only four A site hydride ligands while other complexes include four A site hydride ligands and a B site hydride ligand. Three of the complexes in Table 2 are cations while the remaining complexes are neutral molecules. The ancillary ligands that stabilize these rhenium(V) polyhydride complexes are diverse and include: monodentate tertiary phosphines, a tetradentate ligand with three phosphine centers and an amine, mono- or bidentate phosphite ligands, aliphatic or aromatic amines, a nitrile, or an iodide. The molecular motions consistent with pseudorotational-like behavior that are included in Table 2 are: amine ligand motions (coalescence of ^1^H-{^31^P} NMR resonances for the 3 and 5 protons of an aromatic amine, interconversion of *E* and *Z* isomers by ^31^P-{^1^H} NMR or by ^1^H-{^31^P} NMR, steric inversion of diastereotopic phosphorus atoms by ^31^P-{^1^H} NMR) [33,34,37,65], exchange of a set of three identical phosphorus atoms by ^31^P-{^1^H} NMR [34], or exchange of a set of four A site hydride ligands by ^1^H-{^31^P} NMR spectroscopy [10,33,34,37,58,59,65].

The energy barriers that can be associated with a pseudorotation or pseudorotation-like rearrangement range from 9.1 to 12.9 kcal/mol. The average energy barrier is 10.7 kcal/mol and the standard deviation for the set of values is 1. The rate constant values used for the Eyring equation were estimated from line shape fitting analyses for a range of resonances in dynamic NMR spectra and the energy barriers that appear in Table 2 were all calculated for a temperature of 260 K. Table 2 shows no easily discernible relationship that correlates the size of the energy barrier for a pseudorotation-like rearrangement and some property of each rearranging complex. Table 2 also includes estimates of the enthalpies of activation and entropies of activation for each dynamic process that can be associated with a pseudorotation or pseudorotation-like rearrangement of a complex. The estimates of the enthalpy or entropy of activation fall into two categories: (1) smaller values for the enthalpy of activation and negative entropy of activation and (2) larger values of the enthalpy of activation and positive entropy of activation. The first category of complexes with negative entropies of activation has an average value for ΔH^‡^ of 7.1 kcal/mol with a standard deviation of 1.4. The average ΔS^‡^ value for these same complexes is −15.7 cal/mol∙K with a standard deviation of 7.6. The second category of complexes with a positive entropy of activation has an average value for ΔH^‡^ of 14.5 kcal/mol with a standard deviation of 1.7. The average ΔS^‡^ value for these same complexes is 15.7 cal/mol∙K with a standard deviation of 6.9. The values of ΔH^‡^ and ΔS^‡^ found for the pseudorotation or pseudorotation-like rearrangement of these complexes only have meaning if the rearrangement process is bimolecular. Given the considerable evidence of dihydrogen bonding, hydrogen bond activation, proton exchange between solute molecules and hydride ligands, and even direct hydride ligand transfer between rhenium polyhydride complexes, the notion of a second molecule from the solution interacting with a rhenium polyhydride complex to affect a pseudorotation-like rearrangement of the rhenium polyhydride complex appears to be entirely reasonable.

Several reports describe interactions between solute molecules and rhenium polyhydride complexes [25,26,28,29,31,33,37,63,65,69,74,75,76,77]. A particularly relevant report by Bianchini and coworkers, albeit for a rhenium(III) trihydride complex rather than a rhenium(V) polyhydride complex, estimates ΔH^o^ = −5.9 ± 0.3 kcal/mol and ΔS^o^ = −13.5 ± 0.5 cal/mol∙K for the formation of an adduct between ReH_3_(NP_3_) (NP_3_ = tris[2-(diphenylphosphanyl)ethyl]amine) and 2,2,2-trifluoroethanol [59]. Bianchini’s report describes the adduct as an attraction between the hydroxyl proton of the 2,2,2-trifluoroethanol and a lone pair of electrons on the rhenium center. A similar adduct formation between the lone pair of electrons on a rhenium(V) polyhydride center and a proton of a solute molecule, such as adventitious water [37], may lead to a transition state of higher symmetry that then dissociatively relaxes to affect a pseudorotation-like rearrangement to either of two structural ground states. If the pseudorotation-like rearrangement is indeed bimolecular, then the complexes listed in Table 2 with positive entropies of activation would have the dissociative step as the highest energy step in their rearrangement pathway.

### 6.3. Turnstile Exchange of Rhenium(V) Polyhydride Complexes

The turnstile exchange of a pair of equivalent A site hydride ligands and an adjacent B site hydride ligand is likely to occur for all rhenium polyhydride complexes that include such a hydride ligand configuration. Complexes without a relatively rigid chelating ligand have reported values of ΔH^‡^ that range from 8.7 to 12.3 kcal/mol, with a mean of 10.4 kcal/mol and a standard deviation of 1.3. The values of ΔS^‡^ for such complexes range from −5.7 to 9.3 cal/mol∙K, with a mean of 3.5 cal/mol∙K and a standard deviation of 5.6. The small value of ΔS^‡^ is consistent with the expected unimolecular process for the typical turnstile rearrangement of three hydride ligands. Values of ΔG^‡^_260_ for such a turnstile rotation range from 9.8 to 10.5 kcal/mol, with a mean value of 10.2 kcal/mol and a standard deviation of 0.2. The turnstile rotation of three adjacent hydride ligands at rhenium(V) polyhydride complexes that do not include a rigid chelating ligand appear to have activation parameters that are driven almost entirely by enthalpy.

A single pair of rhenium(V) polyhydride complexes (ReH_4_[η^2^-(1,2-C_6_H_4_)CH=NCH(Me)Ph] and ReH_4_[η^2^-2-(1,2-C_6_H_4_)py]) have been characterized to have a turnstile rotation and also include a rigid chelating ligand that occupies both an A and a B site on a single nonparallel edge of one trapezoid [34]. The activation parameters for the turnstile exchange of three adjacent hydride ligands in this pair of complexes differ markedly from the turnstile exchange parameters for complexes without a rigid chelating ligand. The energy barrier to the turnstile hydride exchange in complexes supported by a rigid chelating ligand arises almost entirely from a very negative value for the entropy of activation, indicating that the turnstile exchange, in the two complexes of interest, may arise from a bimolecular process.

### 6.4. Pairwise Exchange of Adjacent Hydride Ligands

Line shape fitting of dynamic ^1^H-{^31^P} NMR spectra have produced three reports in which an exchange of inequivalent A site hydride ligands occurs [10,34,58]. The activation parameters for the exchange suggest that the process is essentially neutral with respect to the entropy of activation and the energy barrier arises from the enthalpy of activation.

Line shape fitting of dynamic ^1^H-{^31^P} NMR spectra have produced only one report in which adjacent A site and B site hydride ligands exchange with each other [34]. This exchange has only been observed for rhenium(V) polyhydride complexes that include a relatively rigid chelating ligand that occupies the A and B sites along a single edge of one trapezoid within the complex. The exchange of the A and B site hydride ligands along the opposite edge of the same trapezoid proceeds through a process with a significantly negative entropy of activation much like the turnstile hydride ligand exchange for the same complexes. The significant negative entropy may again indicate that such complexes become active towards a pseudorotation-like rearrangement through a bimolecular interaction with another solute molecule.

## 7. Map of the Hydride Ligand Chemical Properties with Respect to the Coordination Site

**Figure 31:**
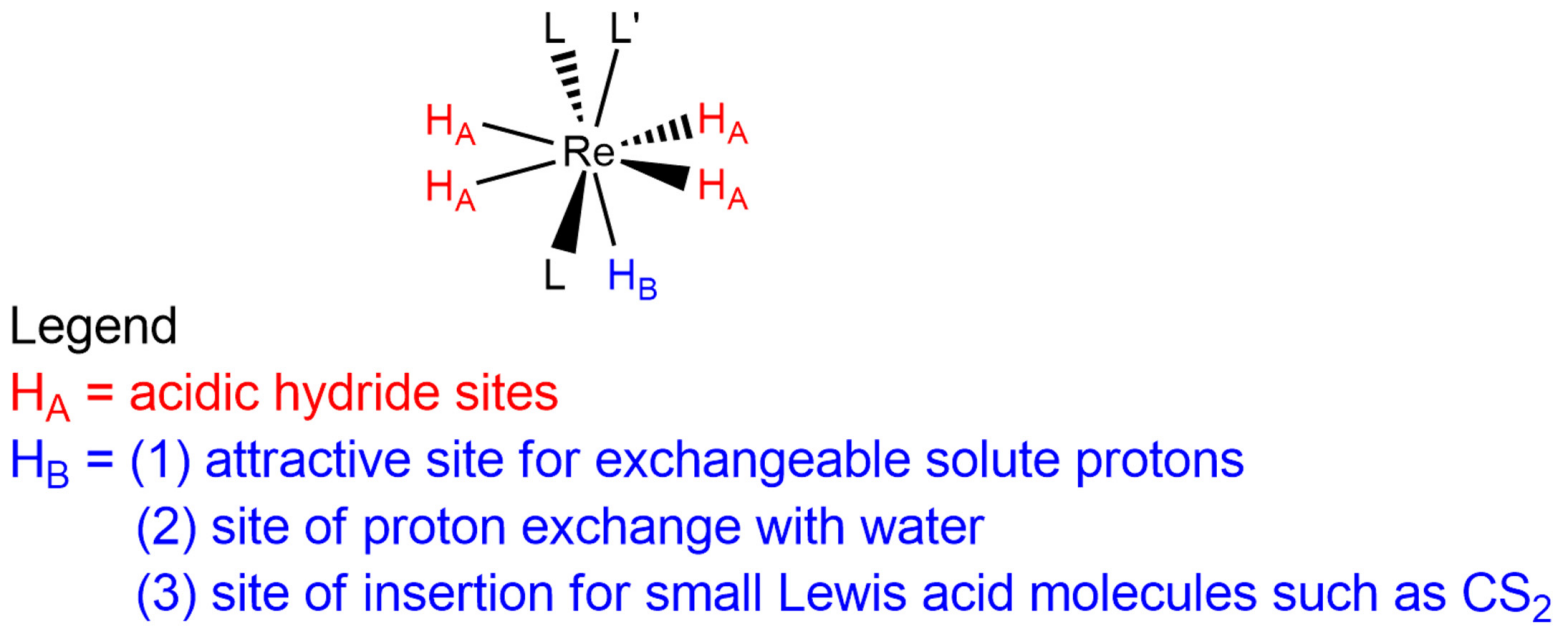
A map of the hydride ligand chemical properties for eight-coordinate rhenium(V) polyhydride complexes.

An understanding of the dynamic processes that occur for rhenium polyhydride complexes is important for both the characterization of new complexes and for the rational design of complexes that will provide specific target properties. Our interest in understanding the dynamic processes of such complexes is a step along the path towards understanding the chemical properties of individual hydride ligands in specific coordination sites of such complexes. The beginnings of a map of the chemical properties versus the coordination sites of hydride ligands in rhenium(V) pentahydride complexes is shown in Figure 31. The map identifies A site hydride ligands as acidic in nature and a unique B site hydride ligand as having a more diverse set of chemical properties.

**Figure 32:**
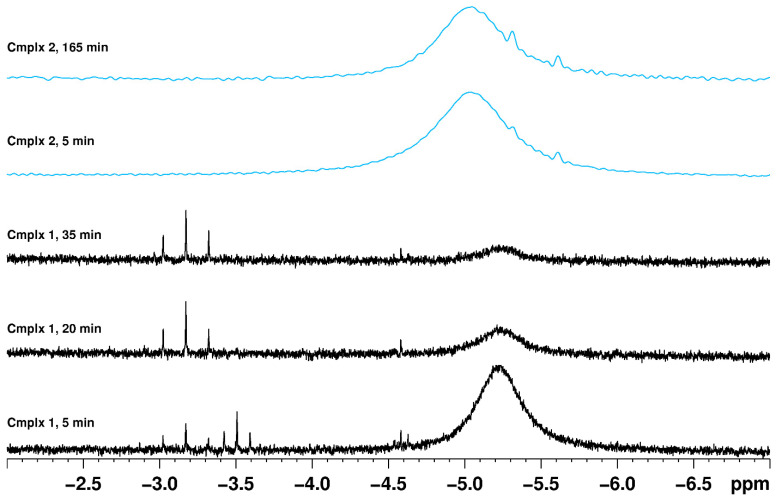
The black ^1^H NMR traces show the hydride region of the spectra, over time, for the complex ReH_5_(PPh_3_)_2_(1,2-ethylene diamine) in a solution of CS_2_:d_6_-acetone (9:1). The blue ^1^H-{^31^P} NMR traces show the hydride region of the spectrum, over time, for the complex ReH_5_(PPh_3_)_2_(2-aminopyridine) in a solution of CS_2_:d_6_-acetone (9:1).

The A site hydride ligands map as acidic in nature based upon the chemical properties of rhenium(V) tetrahydride cations, [(ReH_4_(PPh_3_)_3_L)]^+^. The set of (ReH_4_(PPh_3_)_3_L)^+^ complexes, which include only A site hydride ligands, are readily deprotonated by triethylamine [66]. Rhenium(V) pentahydride complexes, because they are neutral, are not as acidic as the rhenium(V) tetrahydride cations, but we expect that the A site hydride ligands will remain the more acidic hydride ligand site in neutral rhenium(V) pentahydride complexes (rhenium(III) tetrahydride anions are prepared by the deprotonation of neutral rhenium(V) pentahydride complexes). The unique B site hydride ligand in rhenium(V) pentahydride complexes maps as the site that exhibits more diverse chemical properties. The B site hydride ligand has been shown as the site of attraction, in the solid state, for protons bound to nitrogen centers [29,75]. The B site hydride ligand has also been shown to exchange protons with protons from water in a large series of amine-stabilized complexes [33,37,65]. The B site hydride ligand is also the site where insertion of small molecules, such as carbon disulfide, occurs for rhenium polyhydride complexes stabilized by three tertiary phosphine centers. We expect that the specific chemical properties of the B site hydride ligand within a complex will be affected by the nature of the remaining B site ligands, especially the properties of the B site ligand that resides on the same trapezoid as the B site hydride ligand, L’ [8]. In support of our expectation with regards to the effect of the second B site ligand on the chemical properties of the unique B site hydride ligand, we note that ReH_5_(PPh_3_)_2_(amine) complexes are unreactive towards carbon disulfide when stabilized by an aromatic amine ligand while such complexes stabilized by a primary aliphatic amine ligand decompose readily in carbon disulfide (Figure 32).

## 8. Concluding Remarks

The descriptions of the dynamic processes that occur in rhenium polyhydride complexes have advanced from vague, such as the hydride ligands are fluxional in room-temperature solutions, to more specific, such as a turnstile exchange occurs for two A site hydride ligands that are adjacent to a B site hydride ligand in rhenium(V) polyhydride complexes. Aside from the academic interest in identifying and characterizing the dynamic processes in rhenium polyhydride complexes, there are practical aspects that can arise from an understanding of the dynamic processes. The practical aspects of the dynamic properties of rhenium polyhydride complexes, when combined with Morris’ focus on relationships between the properties of ligand sets and the chemical properties of transition metal hydride complexes, can support the rational design of precatalytic rhenium polyhydride complexes [54,94,95]. Such rational design can lead to a better understanding of the catalytic cycles that may occur.

Some practical aspects of the dynamic properties of rhenium polyhydride complexes that relate to the entire set of inner coordination sphere atoms are apparent from the literature. These practical aspects include: (1) when the ligand set makes it feasible to test, all rhenium polyhydride complexes supported by monodentate ligands participate in a pseudorotation or pseudorotation-like rearrangement of all inner coordination sphere atoms in solution at room temperature [1,23,24,30,33,34,35,36,37,49,51,53,54,65,66,68]; (2) the pseudorotation or pseudorotation-like rearrangement of inner sphere atoms can sometimes be slowed or stopped, in room-temperature solutions, by chelating ligands with larger-than-usual ring sizes (>6 member ring), tridentate ligands, or relatively rigid bidentate ligands [8,34,73]; (3) when pseudorotation or pseudorotation-like rearrangement occurs (given an appropriate ligand set and overall symmetry or lack thereof), it can be observed by NMR spectroscopy for both the hydrogen atoms and the heavier atoms [34,35,37,59]; and (4) the characteristics associated with heavy atom rearrangement by a pseudorotation or pseudorotation-like rearrangement should match the characteristics of one of the modes of hydride ligand rearrangement [34,37,59]. For investigators of the chemical properties associated with rhenium polyhydride complexes, the most important aspect related to heavy atom rearrangement is the ability of certain chelating ligands to slow or arrest the room-temperature rearrangement of such complexes. Precatalysts or other such chemical transformation agents centered around rhenium polyhydride complexes are more easily studied in detail when the pseudorotation or pseudorotation-like rearrangement is slow or absent.

More practical aspects of rhenium polyhydride complexes are apparent in the literature with respect to interactions between such complexes and solute molecules. Small molecules with one or more exchangeable protons, in general, have an attractive affinity for rhenium polyhydride complexes [25,26,27,28,29,33,37,74,75,76,77]. This attraction between small molecules with exchangeable protons and rhenium polyhydride complexes may or may not lead to proton exchange between the exchangeable proton and a hydride ligand. At least in the case of rhenium(V) polyhydride complexes, when such proton exchange has been observed, it occurs exclusively at a hydride ligand that resides in a B site [34,37,65]. No exchange between protons from small molecules and A site hydride ligands has been reported for rhenium(V) polyhydride complexes. Several rhenium(VII) polyhydride complexes have been observed to exchange protons between a hydride ligand and an exchangeable proton in a solute molecule [1,3,5,8,31,60,72,73]. The specific site where such exchange occurs in rhenium(VII) complexes has not been identified.

Some aspects of the dynamic processes associated with rhenium polyhydride complexes would benefit from further study. In general, the dynamic processes of nine-coordinate rhenium(VII) polyhydride complexes are less well understood than the eight-coordinate rhenium(V) complexes. Additionally, it is not well understood why some rhenium(VII) complexes exchange hydride ligands for protons of solute molecules while other rhenium(VII) complexes do not. Finally, although many rhenium polyhydride complexes activate carbon-hydrogen bonds, detailed studies of such bond activations are few. Perhaps, as the understanding of the dynamic processes of rhenium polyhydride complexes expands, it will become easier to design studies that examine carbon-hydrogen bond activation at such complexes in better detail.

## Figures and Tables

**Table 1:** Activation parameters for proton exchange between water and the B site hydride ligand of rhenium(V) polyhydride complexes.

Complex	ΔG^‡^_260_ (kcal/mol)	ΔH^‡^ (kcal/mol)	ΔS^‡^ (cal/mol·K)
ReH_5_(PPh_3_)_2_(1,2-ethanolamine) [33]	10.8	14.5	18.5
ReH_5_(PPh_3_)_2_(ethylene diamine) [33]	10.5	13.4	14.4
ReH_5_(PPh_3_)_2_(1,3-diaminopropane) [33]	10.4	13.4	14.9
ReH_5_(PPh_3_)_2_(α,4-dimethylbenzylamine) [37]	8.4	20.7	47.3
ReH_5_(PPh_3_)_2_(2-amino-3-methylbutane) [37]	10.5	13.0	9.4
ReH_5_(PPh_3_)_2_[4-CH3CH(OH)py] [37]	9.9	11.0	4.4

cal/mol·K represents entropy units of calories per [mole × temperature (in units of Kelvin)].

**Table 2:** Thermodynamic parameters for steric inversion-type motions as determined by line shape fitting analysis.

Complex	ΔG^‡^_(260)_ (kcal/mol)	ΔH^‡^ (kcal/mol)	ΔS^‡^ (cal/mol·K)	Resonances Fitted
[ReH_4_(PPh_3_)_3_(NCMe)]PF_6_ [34]	12	8.5	−13.7	^1^H Hydride
	12.9	6.5	−24.8	^31^P
ReH_5_(PPh_3_)_2_(α,4-dimethylbenzylamine) [37]	11.9	6.1	−22.3	^1^H Hydride
	11.8	7.6	−16	^31^P
ReH_5_(PPh_3_)_2_[4-CH3CH(OH)py] [37]	11.8	5.6	−23.7	^1^H Hydride
	11.6	6.7	−18.8	^1^H 3 and 5
ReH_5_(PPh_3_)_2_(2-amino−3-methylbutane) [37]	11.5	5.7	−22.3	^1^H Hydride
ReH_5_(PPh_3_)_2_(*sec*-butylamine) [33]	11.4	9	−9.2	^1^H Hydride
	11.5	6.2	−20.5	^31^P
ReH_4_I(PPh_3_)_3_ [34]	10.9	6.3	−17.6	^1^H Hydride
	11.2	4.9	−24.4	^31^P
ReH_5_(PPh_3_)_2_(1,3-diaminopropane) [33]	11	7.5	−13.6	^1^H Hydride
ReH_5_[Cy_2_PO(CH_2_)_2_OPCy_2_][P(OEt)_3_] [10]	10.9	14.1	12.5	^1^H Hydride
ReH_5_[Cy_2_PO(CH_2_)_2_OPCy_2_][PPh(OEt)_2_] [10]	10.8	13.1	8.9	^1^H Hydride
ReH_5_(PPh_3_)_2_(1,2-diaminoethane) [33]	10.8	8.9	−7.3	^1^H Hydride
ReH_5_(PPh_3_)_2_(1,2-ethanolamine) [33]	10.6	9.7	−3.6	^1^H Hydride
ReH_5_(PPh_3_)_2_(3-aminopyridine) [33]	10.3	14.4	15.8	^31^P
ReH_5_(PPh_3_)_2_(3-picoline) [33]	10.4	16.9	24.8	^31^P
	10.3	16	22.1	^1^H Methyl
ReH_5_[Cy_2_PO(CH_2_)_2_OPCy_2_][PPh_2_(OEt)] [10]	9.9	12.3	9.3	^1^H Hydride
ReH_5_(PPh_3_)_3_ [33]	9.3	6.9	−9.2	^31^P
[ReH_4_(η^4^-tris{2-(diphenylphosphanyl)ethyl}amine]^+^ [59]	9.1	8	−2.9	^1^H Hydride

cal/mol·K represents entropy units of calories per [mole × temperature (in units of Kelvin)].

## Data Availability

Data sharing not applicable. No new data were created or analyzed in this study.
